# The N-cadherin interactome in primary cardiomyocytes as defined using quantitative proximity proteomics

**DOI:** 10.1242/jcs.221606

**Published:** 2019-02-11

**Authors:** Yang Li, Chelsea D. Merkel, Xuemei Zeng, Jonathon A. Heier, Pamela S. Cantrell, Mai Sun, Donna B. Stolz, Simon C. Watkins, Nathan A. Yates, Adam V. Kwiatkowski

**Affiliations:** 1 Department of Cell Biology, University of Pittsburgh School of Medicine, Pittsburgh, PA 15261, USA; 2 Biomedical Mass Spectrometry Center, University of Pittsburgh Schools of the Health Sciences, Pittsburgh, PA 15261, USA; 3 University of Pittsburgh Cancer Institute, Hillman Cancer Center, Pittsburgh, PA 15232, USA

**Keywords:** CDH2, N-cadherin, Cardiomyocyte, Adherens junction, Intercalated disc, Proteomics, Interactome

## Abstract

The junctional complexes that couple cardiomyocytes must transmit the mechanical forces of contraction while maintaining adhesive homeostasis. The adherens junction (AJ) connects the actomyosin networks of neighboring cardiomyocytes and is required for proper heart function. Yet little is known about the molecular composition of the cardiomyocyte AJ or how it is organized to function under mechanical load. Here, we define the architecture, dynamics and proteome of the cardiomyocyte AJ. Mouse neonatal cardiomyocytes assemble stable AJs along intercellular contacts with organizational and structural hallmarks similar to mature contacts. We combine quantitative mass spectrometry with proximity labeling to identify the N-cadherin (CDH2) interactome. We define over 350 proteins in this interactome, nearly 200 of which are unique to CDH2 and not part of the E-cadherin (CDH1) interactome. CDH2-specific interactors comprise primarily adaptor and adhesion proteins that promote junction specialization. Our results provide novel insight into the cardiomyocyte AJ and offer a proteomic atlas for defining the molecular complexes that regulate cardiomyocyte intercellular adhesion.

This article has an associated First Person interview with the first authors of the paper.

## Abstract (precis)

**Highlighted Article:** Proximity proteomics reveals a specific and specialized N-cadherin (CDH2) interactome along the cell–cell contacts of primary cardiomyocytes.

## INTRODUCTION

Heart function requires mechanical coupling and chemical communication between cardiomyocytes through a specialized adhesive structure called the intercalated disc (ICD). The ICD is formed from three junctional complexes: adherens junctions (AJs) and desmosomes that physically link opposing cardiomyocytes, and gap junctions that electrically couple cardiomyocytes (Ehler, 2016; Vermij et al., 2017; Vite and Radice, 2014). AJs and desmosomes link the actin and intermediate filament (IF) cytoskeletons, respectively, to the ICD and provide structural integrity and mechanical strength to the cell–cell contact. ICD formation requires multiple adhesion, cytoskeletal and signaling proteins, and mutations in these proteins can cause cardiomyopathies (Ehler, 2018). However, the molecular composition of ICD junctional complexes remains poorly defined.

The core of the AJ is the cadherin–catenin complex (Halbleib and Nelson, 2006; Ratheesh and Yap, 2012). Classical cadherins are single-pass transmembrane proteins with an extracellular domain that mediates calcium-dependent homotypic interactions. The adhesive properties of classical cadherins are driven by the recruitment of cytosolic catenin proteins to the cadherin tail, with p120-catenin (CTNND1) binding to the juxta-membrane domain and β-catenin (CTNNB1) binding to the distal part of the tail. β-Catenin, in turn recruits αE-catenin (CTNNA1) to the cadherin–catenin complex. α-Catenin is an actin-binding protein and the primary link between the AJ and the actin cytoskeleton (Drees et al., 2005; Pokutta et al., 2014; Rimm et al., 1995; Yamada et al., 2005). In mice, loss of AJ proteins in the heart – N-cadherin (CDH2), β-catenin, αE-catenin or αT-catenin (CTNNA3) – causes dilated cardiomyopathy (Kostetskii et al., 2005; Li et al., 2012b, 2005; Sheikh et al., 2006). Mutations in αT-catenin, an α-catenin homolog expressed predominantly in the heart and testes, and the β-catenin homolog plakoglobin (JUP) have been linked to arrhythmogenic right ventricular cardiomyopathy (Li et al., 2012a; van Hengel et al., 2013), as have disruptions in β-catenin signaling (Garcia-Gras et al., 2006).

The AJ is best understood in the context of epithelia, where it regulates intercellular adhesion, cell motility and polarity (Garcia et al., 2018; Padmanabhan et al., 2015). The AJ can both sense and respond to mechanical force (Charras and Yap, 2018; Hoffman and Yap, 2015), though the molecular mechanism remains largely undefined. In epithelia, the AJ associates with a panoply of proteins that regulate adhesion, signaling and protein turnover. Recent proteomic studies have begun to define the cadherin interactome and have offered new insight into the molecular complexes that regulate AJ biology in epithelia (Guo et al., 2014; Van Itallie et al., 2014). Yet, it is unclear whether these complexes are shared between cell types or whether specific proteins are recruited to AJs to meet specific physiological needs. For example, in cardiomyocytes the AJ is thought to anchor myofibrils to the ICD to transmit force between cells. Whether and how the cardiomyocyte AJ proteome is tuned to meet the mechanical demands of myocyte contraction is not known.

Here, we describe efforts to define the molecular complexes associated with N-cadherin at cardiomyocyte cell–cell contacts. We use a combination of light and electron microscopy to reveal that primary neonatal cardiomyocytes assemble junctional complexes along developing intercellular contacts with structural hallmarks reminiscent of the ICD in adult heart tissue. We show that cardiomyocyte AJ proteins are stable, with dynamics similar to epithelia. We use proximity proteomics to identify N-cadherin-associated proteins along cardiomyocyte cell–cell contacts. We define a robust repertoire of interactors, primarily comprising adaptor and adhesion proteins unique to cardiomyocytes. Our results offer novel insight into the critical adhesion complexes that connect cardiomyocytes and provide a proteomic platform for deciphering how molecular complexes are organized to regulate cardiomyocyte adhesion and cellular organization.

## RESULTS

### Organization of primary cardiomyocyte intercellular contacts

**Fig. 1.**
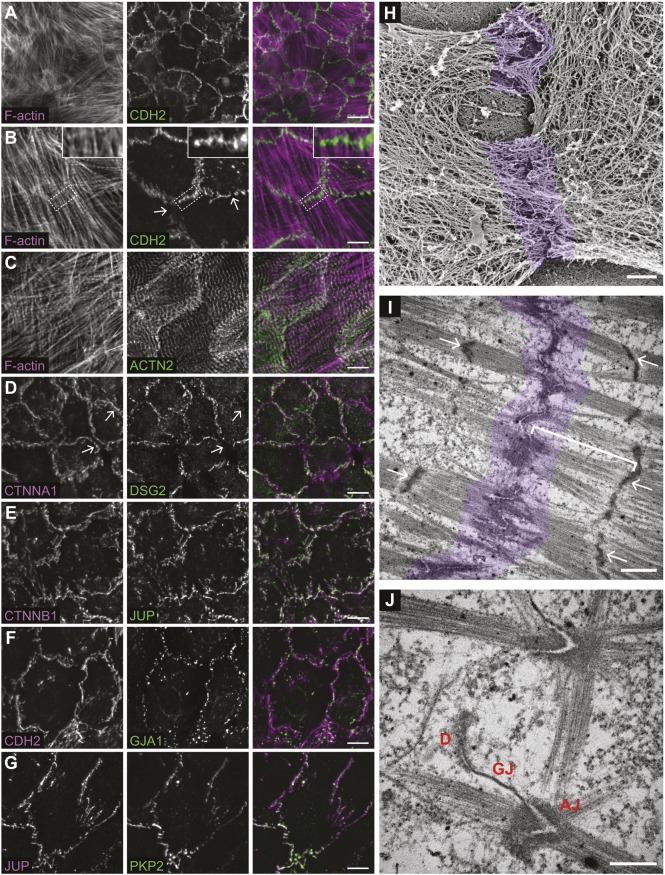
**Cardiomyocyte cell–cell contact organization and architecture.** (A–G) Mouse neonatal cardiomyocytes plated to confluency, fixed 48–72 h post-plating and stained for: F-actin and CDH2 (A,B), F-actin and ACTN2 (C), CTNNA1 and DSG2 (D), CTNNB1 and JUP (E), CDH2 and GJA1 (F) and JUP and PKP2 (G). Individual channels and merge shown. All images are maximum projections of deconvolved Z-stacks. In B, the white arrows mark gaps in CDH2 staining along contacts and the inset is a magnification of the boxed contact that highlights myofibril integration at contacts. In D, white arrows mark CTNNA1-positive, DSG2-negative contacts. (H) Platinum replica electron microscopy image of two connected cardiomyocytes. (I,J) Thin section electron microscopy images of cardiomyocyte cell–cell junctions. In H,I, the cell–cell contact is highlighted in purple. In I, white arrows point to Z-discs and the white bar defines a membrane-proximal sarcomere. In J, desmosome (D), gap junction (GJ) and adherens junction (AJ) are labeled. Scale bars: 20 µm in A; 10 µm in B–G; 500 nm in H; 1 µm in I; 500 nm in J.

Primary cardiomyocytes isolated from rodent neonates retain the ability to establish cell–cell contacts in culture (Franke et al., 2007; Goncharova et al., 1992). Neonatal cardiomyocytes from mice are also amenable to transient transfection and adenoviral infection (Ehler et al., 2013). To begin to define the junctional complexes at newly formed contacts in neonatal cardiomyocytes, we first examined the recruitment of endogenous CDH2, the core of the AJ. Mouse neonatal cardiomyocytes were isolated from postnatal day (P)0–P2 pups and plated on isotropic collagen I substrates at high density to promote intercellular interactions. After 2–3 days in culture, neonatal cardiomyocytes had established CDH2-positive contacts around much of their perimeter (Fig. 1A,B). Myofibril formation is evidenced by the periodic, sarcomeric organization of the Z-line marker α-actinin (ACTN2) (Fig. 1C). Notably, CDH2 localization is not uniform along contacts; instead, it is discontinuous (Fig. 1B, white arrows) and often concentrated at sites of myofibril coupling between cells (Fig. 1B, inset).

We then examined the localization of other primary components of the ICD junctional complexes: AJ, desmosomes and gap junctions. As expected, the AJ proteins CTNNA1 (Fig. 1D) and CTNNB1 (Fig. 1E) showed patterns of localization identical to CDH2. Two desmosome proteins, JUP and plakophilin 2 (PKP2), also showed patterns of localization nearly identical to the AJ (Fig. 1E,G). JUP can bind directly to classical cadherins (Choi et al., 2009) and PKP2 can bind to CTNNA3, where it is thought to link the AJ to intermediate filaments at hybrid junctions when AJ and desmosome proteins are mixed in mammalian hearts (Borrmann et al., 2006; Goossens et al., 2007). The desmosomal cadherin desmoglein 2 (DSG2) also concentrated at cell–cell contacts, but its localization was more restricted than the AJ, with some contacts lacking DSG2 (Fig. 1D, white arrows mark CTNNA1 positive, DSG2 negative contacts). Finally, the gap junction protein connexin 43 (GJA1) showed a punctate pattern of localization along contacts (Fig. 1F). Thus, the primary ICD junctional complexes are recruited to neonatal cardiomyocyte cell–cell contacts.

Next, we sought to define the actin architecture at contacts. We used platinum replica electron microscopy (PREM) to examine actin organization with single-filament resolution (Svitkina, 2017). Cardiomyocytes are enshrouded by a dense cortical cytoskeleton that masks the underlying myofibril network and its association with junctional complexes (Fig. 1H, junctions highlighted in purple). We then used thin section transmission electron microscopy (TEM) to examine junction architecture. Thin section TEM revealed myofibrils coupled along electron-dense contacts (Fig. 1I,J; junction highlighted in purple). The contacts are highly convoluted, and many nascent junctions adopt a chevron-like appearance (Fig. 1J). In addition to AJs, desmosomes and gap junctions are also observed (Fig. 1J), consistent with the results obtained using immunostaining (Fig. 1D,F). Importantly, the junctional topology of cultured cardiomyocytes is similar to that observed in adult hearts, where the angled junctions may help to balance shear versus tensile stresses during contraction (Bennett et al., 2006). Taken together, we conclude that neonatal cardiomyocytes build junctional complexes with many of the organizational and structural hallmarks of adult heart tissue.

### Adherens junction protein dynamics

**Fig. 2.**
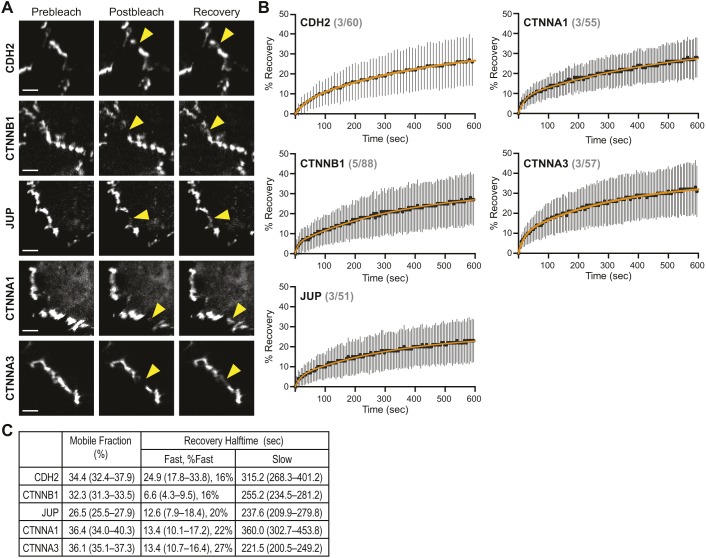
**Adherens junction protein dynamics at cardiomyocyte cell–cell contacts.** (A) Representative pre-bleach, post-bleach and recovery images from FRAP studies of cells expressing GFP-tagged CDH2, CTNNB1, JUP, CTNNA1 and CTNNA3. Yellow arrowheads mark the FRAP region along a cell–cell contact. Scale bars: 50 µm. (B) Plots of mean±s.d. FRAP recovery fraction over time. The data were fit to a double exponential curve (orange line). Values in gray show number of experiments/FRAP contacts quantified for each protein. (C) Summary of the mobile fraction (as percentages) and recovery halftimes (fast and slow pools). The percentage of the fast pool also listed.

We next examined the dynamics of CDH2 and associated catenin proteins in cardiomyocytes. GFP-tagged CDH2, CTNNB1, JUP, CTNNA1 and CTNNA3 were individually transfected into cardiomyocytes. All fusion constructs localized to cell–cell contacts, as expected (Fig. 2A). Protein dynamics were measured using fluorescent recovery after photobleaching (FRAP) in dense cells that had been plated for 48–72 h (Fig. 2A). Fluorescence recovery over ten minutes was quantified, plotted and fit to a double exponential curve (Fig. 2B). The mobile fractions of junctional CDH2 (34.4%), CTNNB1 (32.3%), JUP (26.5%), CTNNA1 (36.4%) and CTNNA3 (36.1%) were all similar to each other (Fig. 2C). Notably, these fractions were nearly identical to those observed in epithelial cells (Yamada et al., 2005) indicating that the majority (approximately two thirds) of cadherin–catenin complexes are immobile components of the AJ plaque.

We then assessed the recovery rates of the mobile fractions for both the fast and slow pools (Fig. 2C). For the cytoplasmic catenins, the fast pool recovery halftimes (6.6–13.4 s) could reflect an unbound, cytosolic population of protein near cell contacts. Alternatively, the pool could be caused by photoswitching (Mueller et al., 2012). The fast pool recovery of the transmembrane CDH2 (24.9 s) likely represents photoswitching because we do not expect diffusion of new CDH2 during this initial time frame. Importantly, the fast pool for all components is relatively small (16–27%) and the slow pool represents the dynamics of the majority of the junction population. Here, the half-times of fluorescence recovery were also similar: CDH2 (315.2 s), CTNNB1 (255.2 s), JUP (237.6 s), CTNNA1 (360.0 s) and CTNNA3 (221.5 s). This reflects the tight associations between core components of the cadherin–catenin complex (Pokutta et al., 2014) and suggests that the multiprotein complex is exchanged as a unit along contacts. While E-cadherin (CDH1), CTNNB1 and CTNNA1 were found to have similar rates of recovery at epithelial cell–cell contacts, the rates were approximately an order of magnitude faster, in the realm of 26–40 s (Yamada et al., 2005). It is unclear what underlies this difference, but it could reflect differences in CDH2-mediated *trans* interactions (Katsamba et al., 2009; Vendome et al., 2014) or stronger association with the actin cytoskeleton. Taken together, our results suggest that cardiomyocytes form stable AJs with properties similar to epithelia.

### CDH2–BioID2 biotinylates proteins at cardiomyocyte cell–cell contacts

**Fig. 3.**
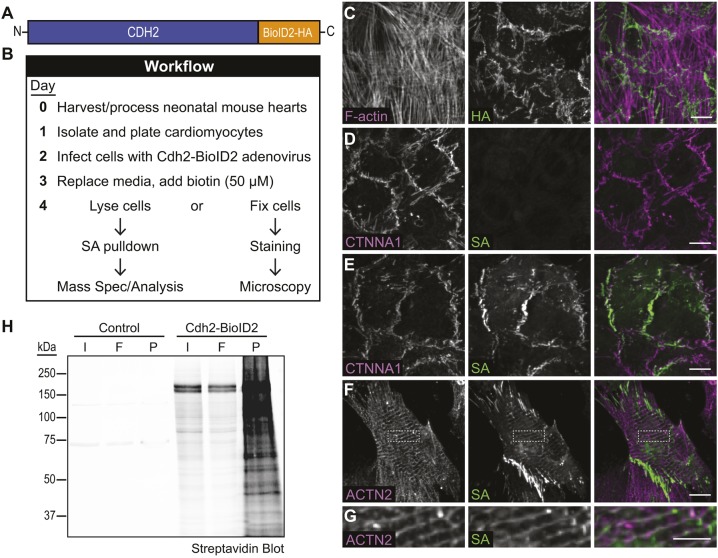
**CDH2–BioID2 localizes to cell contacts and labels junctional proteins.** (A) Schematic of CDH2–BioID2 fusion. (B) Experimental workflow for infecting primary cardiomyocytes, labeling with biotin, and protein fixation or isolation. (C) CDH2–BioID2-infected cardiomyocytes were stained for F-actin (magenta in merge) and HA (green in merge) to identify the HA-tagged fusion construct. (D,E) Uninfected (D) and CDH2–BioID2-infected (E) cardiomyocytes were stained for CTNNA1 and labeled with a streptavidin (SA) conjugated to CY3 to identify biotinylated proteins. (F,G) CDH2–BioID2-infected cardiomyocytes stained for ACTN2 and biotin (SA). G is a high-magnification image of the boxed region in F, highlighting biotinylated proteins along Z-lines. All images in C–G are maximum projections of deconvolved *z*-stacks. (H) Streptavidin western blot of pulldowns from control and CDH2–BioID2-infected cardiomyocytes. Initial material (I), flow through (F) and precipitated material (P) marked. Scale bars: 10 µm in C–F; 5 µm in G.

Given the unique structural and mechanical qualities of cardiomyocyte cell–cell contacts, we next sought to define the molecular complexes along the junctional membrane. We used proximity proteomics to identify proteins near CDH2 by fusing the biotin ligase BioID2 (Kim et al., 2016a) to the C-terminal tail of CDH2 (Fig. 3A). This technique has been used with success to define the CDH1 interactome in epithelia (Guo et al., 2014; Van Itallie et al., 2014) and define CTNNA1 force-dependent molecular interactions (Ueda et al., 2015). We cloned the CDH2–BioID2 fusion into an adenoviral expression system, creating an adenovirus expressing CDH2–BioID2 that would allow us to infect primary cardiomyocytes and express low levels of CDH2–BioID2 for imaging and protein analysis (Fig. 3B). We were able to reproducibly infect >90% of cardiomyocytes at a low multiplicity of infection (MOI). The CDH2–BioID2 fusion localized to cell–cell contacts (HA stain, Fig. 3C), similar to endogenous CDH2 (Fig. 1A,B). Importantly, when biotin (50 µM) was added to the culture, CDH2–BioID2 was seen to label proteins along cell–cell contacts (SA stain in Fig. 3E; compare to uninfected control in Fig. 3D). Biotin addition and concomitant labeling did not disrupt cell–cell contacts (Fig. 3E) and optimal biotinylation was achieved after 24 h (Fig. S1). In addition to the prominent junction labeling, a smaller population of biotinylated proteins was observed at Z-discs (Fig. 3F,G). Finally, we were able to precipitate biotinylated proteins from lysates of infected cells cultured with biotin (Fig. 3H). Thus, CDH2–BioID2 localizes to cardiomyocyte cell–cell contacts and labels proximal proteins that can be isolated for proteomic analysis.

### Quantitative proximity proteomics reveals the cardiomyocyte CDH2 interactome

We used quantitative mass spectrometry (MS) to define the CDH2 interactome. For each replicate, 4×10^6^ cells were infected with CDH2–BioID2 adenovirus, biotin was added to the media and the cells were harvested following the workflow in Fig. 3B. Uninfected control samples were treated identically to CDH2–BioID2 samples (i.e. 50 µM biotin added 48 h post-plating and cells harvested 24 h after biotin addition). Six CDH2–BioID2 replicates and six control replicates were collected and analyzed.

**Fig. 4.**
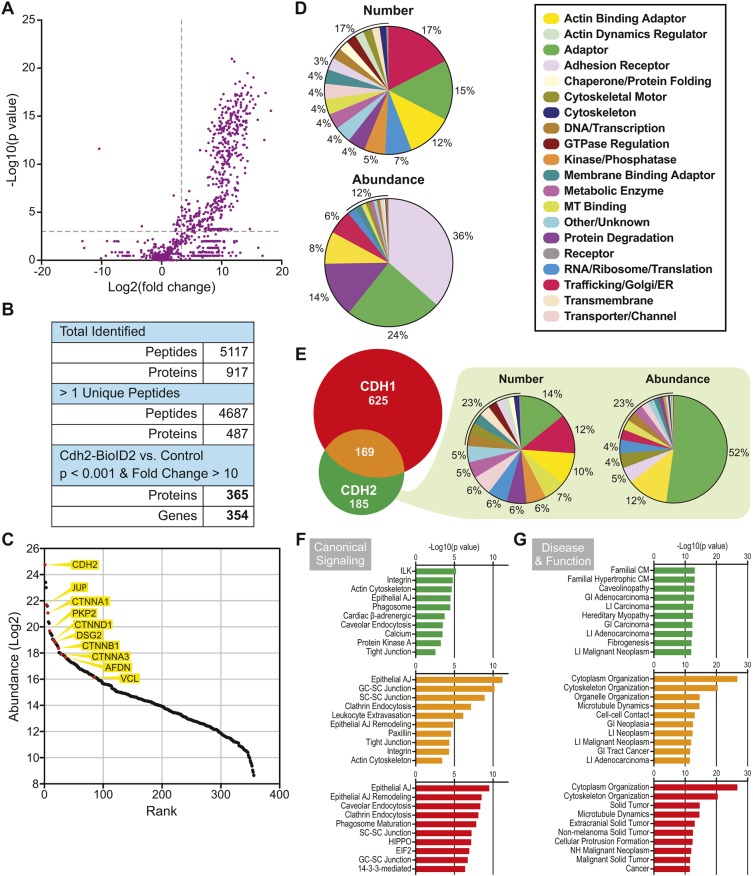
**Quantitative mass spectrometry identifies CDH2 interactome.** (A) Plot of *P*-value (−log_10_) versus fold-change (log_2_) (described in Materials and Methods) of identified proteins. Dashed gray lines mark *P*=0.001 (*y* axis) and fold-change=10 (*x* axis). (B) Summary of numbers of identified peptides and proteins at each stage of further condition stringency. (C) Rank plot of abundance (iBAQ mass, log_2_). Proteins of interest are marked as red circles and labeled. (D) Protein distribution by assigned category based on number (top pie chart) or abundance (iBAQ) (bottom pie chart). (E) Venn diagram of CDH2 interactome in cardiomyocytes (green) versus CDH1 interactome from epithelial cells (red). 169 proteins are shared (orange). Distribution of the CDH2-only pool (minus CDH2, 184 proteins) based on number (left) or abundance (right). (F,G) IPA enrichment analysis of CDH2-only (green), CDH2/CDH1-shared (orange) and CDH1-only (red) groups in canonical signaling pathways (F) or disease and function (G). Abbreviations: AJ, adherens junction; CM, cardiomyopathy; GC, germ cell; GI, gastrointestinal; LI, large intestine; NH, non-hematologic; SC, Sertoli cell.

MS sample analysis revealed a total of 5117 peptides from 917 proteins (Fig. 4A,B). The mean coefficient of variance for the CDH2–BioID2 replicates was ∼30% (Fig. S2). When single unique peptides were excluded, the list was reduced to 4687 peptides from 487 proteins (Fig. 4B). To define CDH2–BioID2 enriched proteins, we established thresholds of fold change≥10 and *P*<0.001 (Fig. 4A, dashed grey lines; see Materials and Methods). These thresholds culled the list to a final 365 proteins from 354 genes (Fig. 4B; Table S1).

The relative abundance of these 365 proteins is plotted in Fig. 4C and the 35 most abundant proteins are listed in Table 1. Among the most abundant proteins were core components of the AJ, including CTNNB1, JUP, CTNND1 and CTNNA1. These same proteins were also abundant in the CDH1 interactome (Guo et al., 2014; Van Itallie et al., 2014). The desmosome components DSG2 and PKP2 were also abundant hits, as were CTNNA3 and the α-catenin ligands vinculin (VCL) and afadin (AFDN) (Hazan et al., 1997; Pokutta et al., 2002; Tachibana et al., 2000; Weiss et al., 1998). The abundance of desmosomal proteins DSG2 and PKP2 could reflect the proximity of AJs and desmosomes along developing cardiomyocyte junctions and/or the proposed intermingling of junctional components in hybrid junctions (Franke et al., 2006). The enrichment of VCL and AFDN, two actin-binding proteins that help anchor the AJ to actin (Sawyer et al., 2009; Yonemura et al., 2010), likely reflects the importance of these proteins in connecting the AJ to the myofibril network.

**Table 1.**
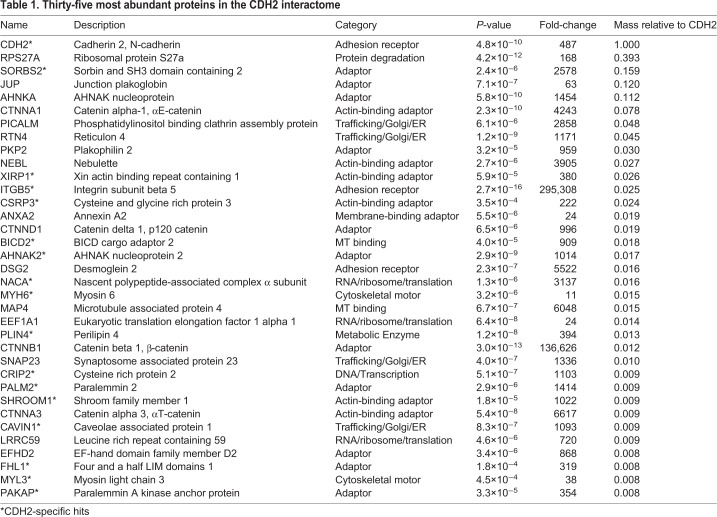
**Thirty-five most abundant proteins in the CDH2 interactome**

### The cardiomyocyte CDH2 interactome is distinct from the epithelial CDH1 interactome

We assigned each of the 354 genes in the CDH2–BioID2 interactome into one of 20 functional categories according to information from Uniprot, GeneCards and Entrez (Fig. 4D), similar to Guo et al. (2014). By number, the categories with the most hits were trafficking/Golgi/ER (17%), adaptor (15%) and actin-binding adaptor (12%). However, when considering protein abundance [represented by intensity-based absolute quantification (iBAQ) values], the top categories were adhesion receptor (36%), adaptor (24%), protein degradation (14%) and actin-binding adaptor (8%) (Fig. 4D). Given the substantial, electron-dense structures built along cardiomyocyte AJs (Fig. 1I,J), the abundance of adaptor proteins and adhesion receptors could function to help couple myofibrils between cardiomyocytes.

We then compared the CDH2–BioID2 hits with the CDH1 interactome from epithelia (Guo et al., 2014; Van Itallie et al., 2014). There are 169 proteins shared between the two interactomes (Fig. 4E) and 185 proteins unique to CDH2 in cardiomyocytes. The distribution of the CDH2-only hits was similar to the entire population (Fig. 4D), with adaptor proteins forming the largest class in number and abundance (Fig. 4E). Actin-binding adaptors, adhesion receptors and cytoskeletal motor proteins were also enriched in the CDH2-only pool (Fig. 4E). By abundance, adaptor proteins (adaptor, actin-binding adaptor and membrane-binding adaptor classes) account for 65% of the CDH2-only pool, highlighting the specialized molecular machinery required for intercellular adhesion in cardiomyocytes.

To gain further insight into the potential similarities and differences between CDH2 and CDH1 interactomes, we performed enrichment analysis using Ingenuity Pathway Analysis (IPA). We examined the CDH2-specific, CDH1-specific and CDH2/CDH1-shared protein sets in canonical signaling, and in disease and function pathways. The CDH1-specific and CDH2/CDH1-shared sets were both enriched for AJ, cell–cell and endocytosis signaling (Fig. 4F; Table S2). In contrast, the CDH2-specific pool showed less enrichment overall, though the emergence of cardiac β-adrenergic and calcium signaling pathways could reflect how the CDH2 interactome is tuned to cardiac function (Fig. 4F; Table S2). The top enriched disease and function pathways for the CDH1-specific and CDH2/CDH1-shared protein sets were cellular organization and cancer-related categories (Fig. 4G; Table S3). In contrast, the CDH2-specific pool was enriched for a variety of cardiomyopathies (Fig. 4G; Table S3). These results suggest that, in cardiomyocytes, CDH2 recruits and organizes unique molecular complexes to regulate cell–cell adhesion and signaling.

### Differential gene expression contributes to the specialized adhesion complexes in cardiomyocytes

**Fig. 5.**
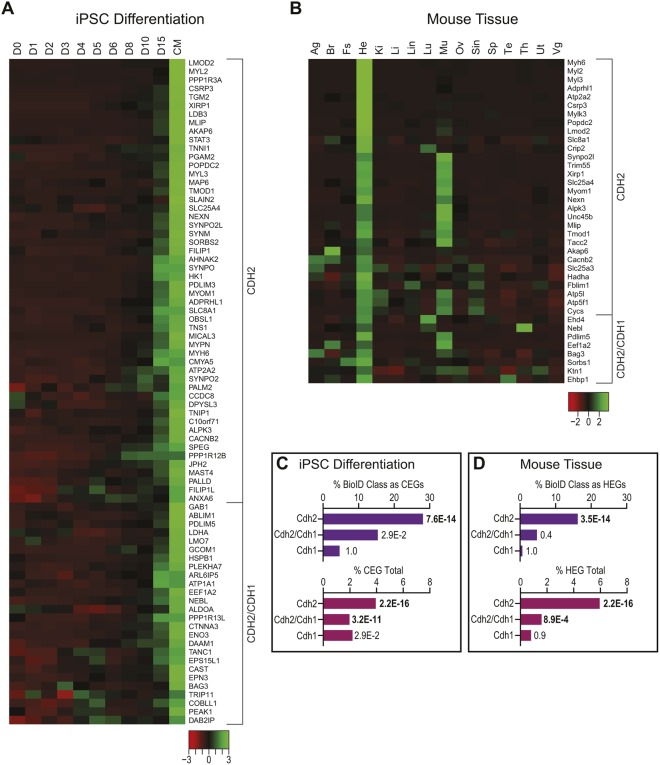
**Differential gene expression contributes to the cardiomyocyte CDH2 proteome.** (A) Heat map of CDH2-only or CDH2/CDH1-shared expression profiles during iPSC differentiation into cardiomyocytes (CM), day 0 (D0) to day 15 (D15). (B) Heat map of CDH2-only or CDH2/CDH1-shared expression profiles in mouse tissues. Ag, adrenal gland; Br, brain; Fs, fore stomach; He, heart; Ki, kidney; Li, liver; Lin, large intestine; Lu, lung; Mu, muscle; Ov, ovary; Sin, small intestine; Sp, spleen; Te, testis; Th, thymus; Ut, uterus; Vg, vesicular gland. (C,D) Top plots: percentage of CDH2-specific, CDH1-specific or CDH2/CDH1-shared BioID class identified as cardiomyocyte enriched genes (CEGs) in human iPSCs (C) or identified as heart enriched genes (HEGs) in mouse tissue (D). Bottom plots: CDH2-specific, CDH1-specific or CDH2/CDH1-shared BioID class in the CEG and HEG categories as a fraction of the total CEG (C) and HEG (D) populations. *P*-value of Fisher's exact test shown to right of each bar. Significant values are in bold.

During development, differential gene expression plays an essential role in establishing cell identity and function. Underlying their specialized role in cardiac contraction, cardiomyocytes express a unique set of genes. To determine whether differential gene expression contributes to the CDH2 interactome, we identified cardiomyocyte- or heart-enriched genes in gene expression profiling data and compared these enriched genes to the CDH2-specific, CDH1-specific and CDH2/CDH1-shared protein sets. We identified 1319 cardiomyocyte-enriched genes (CEGs) from RNA sequencing (RNA-seq) data collected at 11 points during the differentiation of human induced pluripotent stem cells (hiPSCs) to cardiomyocytes (Tompkins et al., 2016). CEGs comprised 22% (78/354) of the CDH2 interactome. Comparative analysis revealed that 52 CEGs were unique to CDH2 (Fig. 5A), representing 28.1% (52/185) of the CDH2 hits (Fig. 5C). In contrast, the number of CEGs present in the CDH1 or CDH2/CDH1 sets was lower, representing just 4.6% and 15.3%, respectively, of the hits for each class (Fig. 5C). We also calculated the percentage of CDH2-specific, CDH1-specific and CDH2/CDH1-shared CEGS in the total CEG pool (1319 CEGs). Fisher's exact test indicated that CEGs were highly enriched in the CDH2 and CDH2/CDH1 sets, but not the CDH1 set (Fig. 5C).

We also analyzed tissue-enriched genes from adult mice. Using RNA-seq data from mouse tissues (Li et al., 2017), we identified 504 heart-enriched genes (HEGs). HEGs comprised 10.7% (38/354) of the CDH2 interactome. Of those, 30 HEGs were present in the CDH2-specific set, representing 16.1% of the CDH2 hits and 6.0% of total HEGs (Fig. 5B,D). HEGs were highly enriched in the CDH2 and CDH2/CDH1 sets, but not the CDH1 set (Fig. 5D). Similar results were observed in gene expression data from human tissues (Fig. S3). Together, these results suggest that cardiomyocyte and heart signature gene expression contribute significantly to the CDH2 interactome. Nonetheless, these enriched genes contribute to ∼10–20% of the CDH2 interactome. The remaining 80–90% of the CDH2 interactome reflects distinct recruitment and organization at the protein level. Thus, while differential gene expression is a significant contributor to interactome identity, the primary driver of AJ specialization in cardiomyocytes is the recruitment of universal adaptor proteins to build specific, multiplex protein complexes.

### CDH2 interactome protein network

**Fig. 6.**
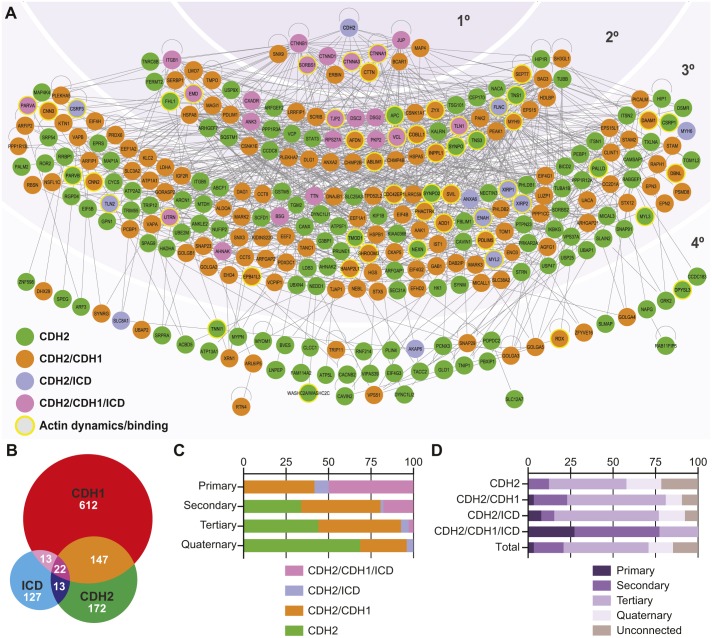
**Cardiomyocyte CDH2 interactome.** (A) Interaction network of CDH2 interactome organized into four tiers based using Ingenuity Pathway Analysis. All protein–protein interactions are supported by published, experimental data. Hierarchical classification was done manually around CDH2. Primary interactors bind CDH2 directly. Secondary interactors bind primary interactors but not CDH2. Tertiary interactors bind secondary interactors. Quaternary interactors bind tertiary interactors or to outermost tier proteins. Bottom left legend defines interactome or protein group classification. (B) Venn diagram between CDH2 interactome, CDH1 interactome and ICD curated proteins. (C) Distribution of interactome or protein groups within each interaction tier. (D) Distribution of tier and unconnected proteins within each group and the total collection.

To better understand how molecular complexes could be assembled at cardiomyocyte AJs, and how these complexes might differ from epithelia AJs, we connected and organized the CDH2 interactome (Fig. 6A). We defined a new interactome group – ICD proteins (curated from the human protein atlas; Estigoy et al., 2009) – and compared it to the CDH2 and CDH1 interactomes. The three-way comparison (Fig. 6B) defined four groups of proteins: CDH2, CDH2/CDH1, CDH2/ICD and CDH2/CDH1/ICD (Fig. 6A). All proteins were color-coded to match their assigned group. We then constructed a hierarchical classification with CDH2 at the top (see Materials and Methods for details). All protein–protein interactions were based on published, experimental data. The classification produced four tiers of interactors: 11 primary, 62 secondary, 177 tertiary and 48 quaternary (Fig. 6A). 52 of the CDH2–BioID2 hits could not be connected to any other protein in the network (Table S4). The hierarchal organization reveals that the percentage of CDH2–BioID2 unique hits (green) increases from 0% to 70% as the distance from CDH2 increases, whereas the percentage of CDH2/CDH1 (orange) and CDH2/CDH1/ICD (pink) groups decreases from >90% to 25% (Fig. 6C,D). This suggests that the primary complex (primary and secondary tiers) is largely shared between CDH2 and CDH1, but that specific, specialized interactors are recruited outside (tertiary and quaternary tiers) the primary complex to regulate junction assembly and function in cardiomyocytes. Also noteworthy is the abundance of CDH2 (green) hits versus CDH2/ICD (purple) or CDH2/CDH1/ICD (pink) hits. These green-labeled proteins reflect potentially new, previously unassigned ICD components with potential roles in cadherin and cardiomyocyte adhesion biology.

### Identified adaptor proteins localize to cell–cell contacts

**Fig. 7.**
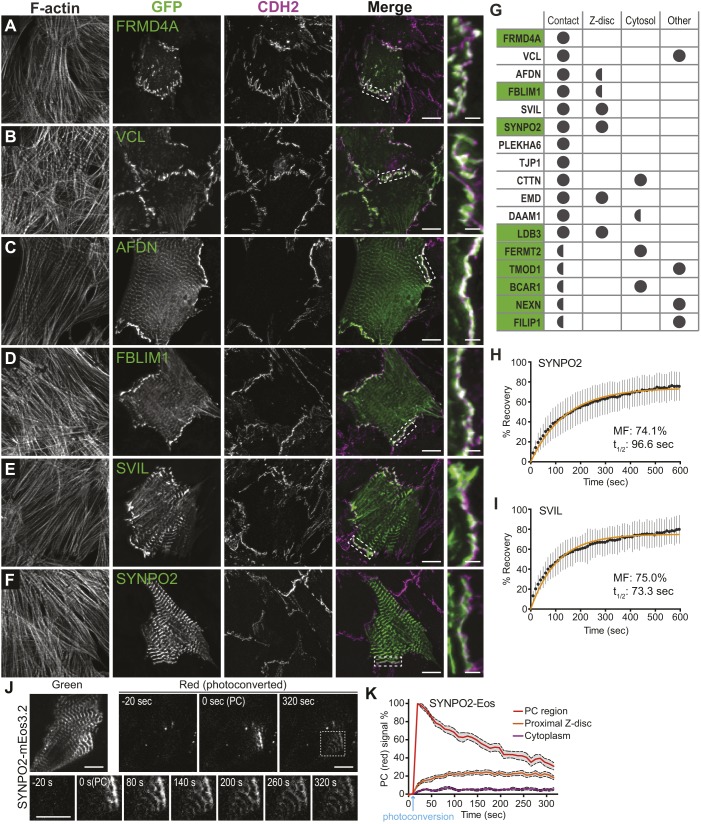
**CDH2 interactome proteins localize to cell–cell contacts and Z-discs.** (A–F) Cardiomyocytes transfected with GFP-tagged CDH2–BioID2 hits as indicated. Cells were fixed 24 h post-transfection and stained for CDH2 and F-actin. All images are maximum projections of deconvolved *z*-stacks. Individual and merged GFP (green) and CDH2 (magenta) channels shown. Far right shows magnification of boxed contact in merge image. (G) Summary of GFP–CDH2–BioID2 interactome localization to cell–cell contacts, Z-discs, cytosol or other. Full circle indicates robust localization, half circle indicates modest localization. Proteins highlighted in green are unique to the CDH2 interactome. Representative images for PLEKHA6, TJP1 (paralog of TJP2), CTTN, EMD, DAAM1, LDB3, FERMT2, TMOD1, BCAR1, NEXN and FILIP1 are shown in Fig. S4. (H,I) Plot of mean±s.d. FRAP recovery fraction over time for SYNPO2 (30 FRAP regions from two independent experiments) and SVIL (18 FRAP regions from two independent experiments) at Z-discs. The data were fit to a single exponential curve (orange line). Mobile fraction (MF) percentage and recovery halftimes (t_1/2_) listed. (J) Dynamics of photoconverted SYNPO2-mEos3.2 in transfected cardiomyocytes. Green channel shows total SYNPO2-mEos3.2 protein. Red channel shows photoconverted protein before activation (−20 s), immediately after photoconversion [0 s (PC)] and after 320 s. Bottom montage shows a magnified view of photoconverted protein (boxed region in top right 320 s panel) over time. (K) Quantification of photoconverted SYNPO2-mEos3.2. Mean percentage of photoconverted protein (red signal) for the photoconverted area (PC region, red line), Z-disc 2–3 μm outside the photoconverted region (Proximal Z-disc, orange line) and cytoplasm 2–3 μm outside the photoconverted region (Cytoplasm, purple line) plotted over time. Dashed lines and gray region around mean define the s.e.m. Time of photoconversion marked with a blue arrow. Data is from 12 photoconverted cells from two independent experiments. Scale bars: 10 µm in A–F,J.

Cardiomyocyte AJs must connect contractile myofibrils, placing unique demands on the proteins that physically connect actin to the cadherin complex. We identified over 100 adaptor proteins in the CDH2 interactome (Fig. 4D, adaptor, actin-binding adaptor and membrane-binding adaptor) and nearly half of these adaptor proteins bind actin or regulate actin dynamics (Fig. 6A, actin-associated proteins highlighted with yellow; Table S1), including the actin-binding proteins VCL and AFDN, both top-ranked hits (Fig. 4C, Table 1). We examined the localization of 27 adaptor and actin-associated proteins by transiently expressing fluorescently-tagged proteins in cardiomyocytes (Fig. 7; Fig. S4). Seventeen of the tested proteins localized to cell–cell contacts (Fig. 7A–F; Fig. S4A; summarized in Fig. 7G). FRMD4A, VCL, AFDN and FBLIM1 localized primarily to cell–cell contacts (Fig. 7A–D). As expected, VCL also localized to cell–substrate contacts (Fig. 7B). AFDN and FBLIM1 were also present at Z-discs (Fig. 7C,D). SVIL and SYNPO2 localized primarily to Z-discs but were also observed colocalizing with CDH2 at contacts (Fig. 7E,F; Fig. S4B). Representative images showing the localization of PLEKHA6, TJP1 (paralog of TJP2), CTTN, EMD, DAAM1, LDB3, FERMT2, TMOD1, BCAR1, NEXN and FILIP1 are shown in Fig. S4A. DBN1 formed filamentous structures along the actin cytoskeleton with limited localization to cell–cell contacts and LNPK localized to the endoplasmic reticulum (Fig. S4A). The remaining eight proteins were primarily cytoplasmic or formed aggregates when overexpressed (Fig. S4A). These could represent false positives, though three of the hits – PARVA, COBLL1 and TLN1 – have been reported to associate with CDH1 (Guo et al., 2014). Notably, 9 of the 17 proteins recruited to cell–cell contacts are unique to the CDH2 interactome (Fig. 7G, highlighted in green) and represent proteins that could promote AJ specialization in cardiomyocytes.

### Dynamic shuttling between AJs and Z-discs

The ICD can be thought of as the terminal Z-disc because, for a membrane-tethered myofibril, the ICD functions as the terminal end of the sarcomere (Fig. 1I). Our localization analysis identified four proteins with strong localization to Z-discs and cell–cell contacts: SYNPO2, SVIL, EMD and LDB3 (Fig. 7E,F; Fig. S4A). In CDH2–BioID2-expressing cardiomyocytes, biotinylated proteins were detected at cell–cell contacts as well as at Z-discs (Fig. 3F,G). We questioned whether proteins could shuttle between the AJ and Z-discs. We first analyzed the dynamics of GFP-tagged SYNPO2 and SVIL using FRAP (Fig. 7H,I). Both proteins were dynamic, with large mobile fractions (∼75%) and fast recovery halftimes (97 s for SYNPO2 and 73 s for SVIL). We then tracked the movement of SYNPO2 further using the photoconvertible protein mEos3.2. Photoconverted SYNPO2–mEos3.2 shuttled from Z-discs or cell–cell contacts to distal Z-discs within minutes (Fig. 7J,K). We speculate that Z-disc proteins are recruited dynamically to the AJ to promote myofibril assembly and integration along cell–cell contacts. Thus, the ICD AJ plays an important role in guiding both cardiomyocyte adhesion and cytoskeleton organization.

## DISCUSSION

Our results provide new details into the architecture of the developing ICD and define the proteins that organize the AJ in cardiomyocytes. This work builds off past studies of the cadherin–catenin interactome in epithelia (Guo et al., 2014; Ueda et al., 2015; Van Itallie et al., 2014) to expand the AJ protein atlas to include the cardiomyocyte, a unique contractile system. Our molecular and proteomics data reveal how the cardiomyocyte AJ recruits a unique set of cytoskeletal, scaffold and signaling proteins to build this critical mechanical junction and guide cardiomyocyte organization and adhesion.

### Core adhesion complexes are conserved

The cadherin–catenin complex is recruited to developing contacts in cardiomyocytes and, not surprisingly, the catenins (CTNNB1, JUP, CTNND1, CTNNA1, CTNNA3) are among the most robust hits in the CDH2 proteomic screen (Table 1). FRAP studies revealed that the complex is largely immobile (approximately one third is mobile) and that the entire complex is exchanged along contacts similar to epithelia (Yamada et al., 2005), though the recovery rate is markedly slower (discussed below). These properties are not unexpected given current AJ dogma. Biochemical studies have demonstrated that β-catenin binds with high affinity to αE-catenin and that the β-catenin–αE-catenin complex binds strongly to the cadherin tail to create a stable complex (Pokutta et al., 2014). The molecular interactions that underlie the core cadherin–catenin complex are likely conserved between epithelia and cardiomyocytes. Consistent with this, CDH1 expression can restore intercellular adhesion and myofibril coupling in cultured CDH2-null cardiomyocytes (Luo and Radice, 2003) and ectopic, cardiac-specific expression of CDH1 in CDH2-null embryos can rescue early heart development (Luo et al., 2001). The ability of CDH1 to rescue basic cadherin functions in CDH2-deficient cardiomyocytes underscores how the core cadherin–catenin complex, and its basic properties, are conserved between epithelia and cardiomyocytes.

The desmosome components DSG2, JUP and PKP2 were also among the more abundant proteins isolated in the proteomic screen (Table 1). DSG2 localization was more restricted than the AJ and appeared to be preferentially localized near sites of myofibril integration (Fig. 1D; C.D.M. and A.V.K., unpublished observations). DSG2 (and desmosome development) may be favored at more stable or mature AJs, consistent with EM analysis (Fig. 1J). Interestingly, recent evidence suggests that CDH1 can recruit DSG2 through direct extracellular *cis* interactions to promote desmosome assembly at nascent contacts in epithelial cells (Shafraz et al., 2018). A similar interaction between CDH2 and DSG2 could promote desmosome assembly along cardiomyocyte junctions. In contrast to DSG2, JUP and PKP2 showed near-identical localization patterns to the AJ (Fig. 1E,G). JUP can bind directly to classical cadherins (Choi et al., 2009) and was a robust hit in CDH1 proteomic screens (Guo et al., 2014; Van Itallie et al., 2014). PKP2, an armadillo protein related to CTNND1, is a multifunctional protein that binds desmosomal cadherins, JUP and desmoplakin (DSP) (Bass-Zubek et al., 2009). We speculate that both JUP and PKP2 may be recruited to AJs during the initial stages of contact formation to promote desmosome assembly, similar to their proposed role in epithelia (Nekrasova and Green, 2013). Note that PKP2 can also bind directly to CTNNA3 and has been proposed to link the AJ to intermediate filaments at hybrid junctions, where AJ and desmosome proteins are mixed in mammalian hearts (Borrmann et al., 2006; Goossens et al., 2007).

### AJ specialization is driven by ancillary adaptor proteins

We identified 365 proteins from 354 genes in the CDH2 interactome. Of these, 169 hits were shared with the CDH1 interactome while 185 hits were unique to CDH2. By abundance, 65% of the CDH2 pool was composed of adaptor proteins (Fig. 4E). Analysis of the protein–protein interactions revealed that shared components occupied the inner tiers of the network whereas CDH2-specific adaptors occupied the outer tiers (Fig. 6A). The assembled interaction network reflects the hierarchy of protein binding required to form the molecular complexes along cardiomyocyte contacts. Critical to these assemblages are the catenin proteins, CTNND1, CTNNB1, JUP, CTNNA1 and CTNNA3. All catenin proteins are known to bind directly to a number of other proteins. For example, in addition to binding CTNNB1/JUP and actin, CTNNA1/CTNNA3 can interact with VCL, AFDN, PKP2 and ZO1/ZO2 (also known as TJP1/TJP2) (Vite et al., 2015). These proteins, in turn, can associate with a wide range of cytoskeletal and signaling proteins. We speculate that the catenins coordinate the organization of molecular complexes at the cardiomyocyte AJ to regulate adhesion and signaling.

Collectively, the primary function of these adaptor proteins is likely to strengthen the mechanical connection between the AJ and the actin cytoskeleton along the ICD membrane. Myofibrils are coupled to AJs at substantial, electron-dense structures (Fig. 1I,J), consistent with a large assemblage of proteins organizing into an adhesive plaque to anchor the contractile filaments. Likewise, the recovery halftime of the cadherin–catenin complex at cardiomyocyte cell–cell contacts is an order of magnitude slower than at epithelial junctions (Fig. 3C) (Yamada et al., 2005), possibly due to stronger connections to the actin cytoskeleton mediated through actin-binding proteins. While adaptor proteins could function to strengthen the physical connection between the cadherin–catenin complex and actin, they might also promote actin and membrane architectures that function to mitigate the forces of contraction. For example, coupling to myofibrils at an angle along the ICD membrane (Fig. 1I,J) may help AJs withstand mechanical force during contraction. Membrane and actin-associated adaptor proteins could regulate the formation and maintenance of these unique ICD junctional topologies.

Roughly 140 curated ICD proteins were not detected in the CDH2 interactome (Fig. 7B), including established CDH2-associated proteins like the gap junction protein connexin 43 (GJA1). While we observed GJA1 along contacts in our cultured cardiomyocytes, the localization was punctate and differed from CDH2. The absence of GJA1 and other curated ICD proteins from our screen could be due to the physical limitations of BioID2-labeling [the range of biotinylation is limited to ∼10 nm, (Kim et al., 2016a)], the absence of surface lysines on a target protein for labeling and/or the maturity of the cell–cell contacts in our system. Alternatively, it could reflect the segregation of molecular complexes along these contacts and highlight the specificity of AJ interactions.

Conversely, only 13 of the 185 CDH2-specific hits are curated ICD proteins. The remaining 172 hits have not been associated with the ICD, thus expanding the atlas of proteins associated with cell adhesion in cardiomyocytes. Note, however, that biotin labeling can occur during many stages of a protein life cycle, thus some hits may not be ICD proteins and instead regulate CDH2 trafficking or degradation. In fact, trafficking/Golgi/ER proteins were a significant fraction (12%, Fig. 4E) of the CDH2-specific hits. Likewise, some hits could be false positives: of the 27 adaptor and actin-associated proteins expressed as GFP-tagged fusions in cardiomyocytes, 8 were cytoplasmic or formed aggregates. However, the GFP tag or expression level could have interfered with the localization of many of these proteins, particularly since some (TLN1, PARVA and COBLL1) are shared with the CDH1 interactome and likely represent conserved interactors (Guo et al., 2014). Also, since proximity labeling occurs in intact, living cells, false interactions that may arise during cell lysis or precipitation are limited (Chen and Perrimon, 2017). Nonetheless, it is essential that any hit be verified using a secondary method (e.g. cell staining or co-precipitation), and we expect that future work will further define and refine the CDH2 interactome.

### The AJ and the Z disc, linked through the myofibril sarcomere

Cardiomyocytes must be coupled to create a functional syncytium and the AJ serves as the mechanical link between myofibrils and the ICD membrane. The contractile unit of the myofibril is the sarcomere whose lateral boundaries are defined by Z-discs where the barbed ends of actin filaments are interdigitated and crosslinked. Z-discs are connected to the lateral membrane (sarcolemma) and the surrounding extracellular matrix by specialized adhesive complexes called costameres. In cardiomyocytes, the AJ functions as the terminal Z-disc for the membrane proximal sarcomere (Fig. 1I). While cardiomyocyte organization almost certainly requires coordination between the AJ and Z-disc/costamere (Bennett et al., 2006), the molecular details remain largely unexplored and undefined. We identified a number Z-disc proteins in our proteomic screen (Table S1), including SVIL (Oh et al., 2003), SYNPO2 (Weins et al., 2001), EMD (Barton et al., 2015; Cartegni et al., 1997), BAG3 (Hishiya et al., 2010), LDB3 (Faulkner et al., 1999), NEBL (Moncman and Wang, 1995), PDLIM3 and PDLIM5 (Zheng et al., 2010), FHL1 (Frank et al., 2006), TTN (Herzog, 2018) and ZYX (Frank and Frey, 2011). We observed that at least four – SVIL, SYNPO2, EMD and LDB3 – localize to both Z-discs and cell–cell contacts (Fig. 7E,F; Fig. S4A). We showed that SYNPO2 and SVIL are dynamic proteins and that SYNPO2 can shuttle between junctions and Z-discs. We speculate that the AJ recruits Z-disc proteins to coordinate myofibril assembly and integration at contacts. Additional studies are expected to reveal how such coordination is regulated at the molecular level.

### The developing ICD in neonatal cardiomyocytes

We took advantage of the innate ability of primary neonatal cardiomyocytes to reestablish cell–cell contacts *in situ* to express tagged AJ proteins and explore their dynamics and label CDH2-associated proteins. Primary neonatal cardiomyocytes plated on isotropic substrates form cell–cell contacts around their entire perimeter (Fig. 1), similar to cardiomyocytes in the developing and perinatal heart (Hirschy et al., 2006). The stereotypical, elongated cardiomyocyte morphology with aligned myofibrils and ICDs restricted to the bipolar ends develops postnatally (Angst et al., 1997; Hirschy et al., 2006; Pieperhoff and Franke, 2007), though the mechanisms of this polarization remain unclear. Thus, while our proteomic results offer a snapshot of the CDH2 interactome at developing cell–cell contacts rather than at mature ICDs, this transitional stage has *in vivo* relevance and these results provide a significant advance in defining the cadherin interactome in cardiomyocytes. In addition, we were able to generate quantitative MS data from a relatively small sample of cultured primary cardiomyocytes. A similar experimental protocol could be used to examine changes in the CDH2 interactome from mutant cardiomyocytes or from cardiomyocytes cultured under varying conditions (e.g. soft versus stiff substrates). Alternatively, it could be used to define the CDH2 proteome in differentiated iPSCs or modified to express in an AAV system to examine the CDH2 proteome *in vivo* during heart development or disease. Future work is expected to build on this newly defined AJ network to provide important insight into how the molecular complexes that regulate AJ function change in response to injury or disease.

## MATERIALS AND METHODS

### Plasmids

Murine Cdh2 in pEGFP-N1 (CDH2-EGFP) was a gift from James Nelson (Stanford University, Stanford, CA, USA). CTNNA3-EGFP was described previously (Wickline et al., 2016). Plasmid pEGFP-C1-rat-l-afadin (AFDN) was gift from Yoshimi Takai (Nakata et al., 2007). Plasmids mEmerald-JUP-N-14 (Addgene 54133), mEmerald-beta-catenin-20 (CTNNB1, Addgene 54017), mEmerald-alpha1-catenin-C-18 (CTNNA1, Addgene 53982), mEmerald-ZO1-C-14 (TJP1, Addgene 54316), mEmerald-Vinculin-23 (VCL, Addgene 54302), mEmerald-Talin-C-18 (TLN1, Addgene 62763), mEmerald-Parvin-N-16 (PARVA, Addgene 54215) and mEmerald-Migfilin-C-14 (FBLIM1, Addgene 54181) were deposited by Michael Davidson. Emerin pEGFP-C1 (EMD, Addgene 61993) was deposited by Eric Schirmer (Zuleger et al., 2011). GFP-cortactin (CTNN1, Addgene 26722) was deposited by Anna Huttenlocher (Perrin et al., 2006). EGFP-supervillin (SVIL, Addgene 13040) was deposited by Elizabeth Luna (Wulfkuhle et al., 1999). Drebin-YFP (DBN1, Addgene 40359) was deposited by Phillip Gordon-Weeks (Geraldo et al., 2008). pEGFP Kindlin2 (FERMT2, Addgene 105305) was deposited by Kenneth Yamada. pHAGE2 Lnp-mCherry (LNPK, Addgene 86687) was deposited by Tom Rapoport (Wang et al., 2016). HA-p62 (SQSTM1, Addgene 28027) was deposited by Qing Zhong (Fan et al., 2010). pDONR223_TRIM55_WT (Addgene 81829) was deposited by Jesse Boehm, William Hahn and David Root (Kim et al., 2016b). MCS-BioID2-HA (Addgene 74224) was deposited by Kyle Roux (Kim et al., 2016a). pAdTrack-CMV (Addgene 16405) was deposited by Bert Vogelstein (He et al., 1998). Sqstm1 and Trim55 were subcloned into pEGFP-N1.

To create the PLEKHA6, FRMD4A, DAAM1, SYNPO2, LDB3, TMOD1, NEXN, FILIP1, CSRP1, DPYSL3, COBLL1 and PHLDB1 constructs, RNA was first isolated and purified from adult mouse heart using an RNeasy Fibrous Tissue Mini Kit (Qiagen) and reverse transcribed to create cDNA using Transcriptor High Fidelity cDNA Synthesis Kit (Roche). Gene specific primers were designed against the 5′ and 3′ ends of each gene to generate full-length clones by PCR. PCR products were cloned directly into pEGFP-N1 (PLEKHA6, SYNPO2, LDB3, NEXN, CSRP1, DPYSL3, COBLL1 and PHLDB1) or pEGFP-C1 (FRMD4A, DAAM1, TMOD1 and FILIP1) to create EGFP fusions. SYNPO2 was also cloned into mEos3.2-N1 (Addgene 54525), deposited by Michael Davidson and Tao Xu (Zhang et al., 2012). Assembled clones were verified by means of sequencing.

### Antibodies

Primary antibodies used for immunostaining were: anti-N-cadherin (1:250, Thermo Fisher Scientific, 33-3900), anti-α-Actinin (1:250, Sigma, A7811), anti-Desmoglein 2 (1:250, Abcam, ab150372), anti-αE-catenin (1:100, Enzo Life Science, ALX-804-101-C100), anti-β-Catenin (1:100, BD Transduction Laboratories, 610154), anti-γ-Catenin (1:100; Cell Signaling Technology, 2309), anti-Connexin-43 (1:100, Proteintech, 15386-1-AP), anti-Plakophilin 2 (1:10, Progen, 651101) and anti-HA (1:100, Sigma, 11867423001). Streptavidin-Cy3 (1:300, Jackson Immunoresearch, 016-160-084) was used to label biotinylated proteins. Secondary antibodies used were goat anti-mouse or anti-rabbit IgG labeled with Alexa Fluor 488, 568 or 647 dyes (1:250, Thermo Fisher Scientific). F-actin was visualized using Alexa Fluor-conjugated phalloidin (1:100–1:250, Thermo Fisher Scientific).

### Cardiomyocyte isolation and culture

All animal work was approved by the University of Pittsburgh Division of Laboratory Animal Resources. Primary cardiomyocytes were isolated from Swiss Webster or Black 6 mouse neonates (P1–P3) as described (Ehler et al., 2013; Wickline et al., 2016). For protein isolation, Swiss Webster-derived cardiomyocytes were plated onto 35 mm dishes (1×10^6^ cells/dish) coated with Collagen Type I (Millipore). For immunostaining, cardiomyocytes were plated onto 35 mm MatTek dishes with 10 mm insets coated with Collage Type I. Cardiomyocytes were plated in plating media: 65% high-glucose DMEM (Thermo Fisher Scientific), 19% M-199 (Thermo Fisher Scientific), 10% horse serum (Thermo Fisher Scientific), 5% FBS (Atlanta Biologicals) and 1% penicillin-streptomycin (Thermo Fisher Scientific). Media was replaced 16 h after plating with maintenance media: 78% high-glucose DMEM, 17% M-199, 4% horse serum, 1% penicillin-streptomyocin, 1 µM AraC (Sigma) and 1 µM isoproternol (Sigma). Cells were cultured in maintenance media for 2–4 days until lysis or fixation.

### Immunostaining and confocal microscopy

Cells were fixed in 4% EM grade paraformaldehyde in PHEM buffer (60 mM PIPES pH 7.0, 25 mM HEPES pH 7.0, 10 mM EGTA pH 8.0, 2 mM MgCl_2_ and 0.12 M sucrose) or PHM (no EGTA) buffer for 10 min, washed twice with PBS and then stored at 4°C until staining. Cells were permeabilized with 0.2% Triton X-100 in PBS for 4 min and washed twice with PBS. Cells were then blocked for 1 h at room temperature in PBS+10% BSA (Sigma), washed 2× in PBS, incubated with primary antibodies in PBS+1% BSA for 1 h at room temperature, washed 2× in PBS, incubated with secondary antibodies in PBS+1% for 1 h at room temperature, washed 2× in PBS and then mounted in Prolong Diamond (Thermo Fisher Scientific). All samples were cured at least 24 h before imaging.

Cells were imaged on a Nikon Eclipse Ti inverted microscope outfitted with a Prairie swept field confocal scanner, Agilent monolithic laser launch and Andor iXon3 camera using NIS-Elements (Nikon) imaging software. Maximum projections of 3–5 µm image stacks were created and deconvolved (3D Deconvolution) in NIS-Elements (Nikon) for presentation.

### FRAP experiments

FRAP experiments were conducted on a Nikon swept field confocal microscope (describe above) outfitted with a Tokai Hit cell incubator and Bruker miniscanner. Actively contracting cells were maintained at 37°C in a humidified, 5% CO_2_ environment. User-defined regions along cell–cell contacts (CDH2, CTNNB1, JUP, CTNNA1 and CTNNA3; Fig. 2) or Z-discs (SYNPO2 and SVIL; Fig. 7) were bleached with a 488 laser and recovery images collected every 5 s or 10 s for 10 min. FRAP data was quantified in ImageJ (NIH) and average recovery plots were measured in Excel (Microsoft). For Fig. 2, FRAP recovery plots represent data from >50 contacts from at least three separate transfections of unique cell preps. For Fig. 7, FRAP recovery plots represent data from 30 (SYNPO2) or 18 (SVIL) Z-discs from two independent transfections of unique cell preps. Curves were either fit to a double exponential formula (Fig. 2) or a single exponential formula (Fig. 7), whichever fit the recovery data the best, to determine the mobile fraction and half time of recovery in Prism (GraphPad).

### Photoconversion experiments

Transfected cardiomyocytes were cultured and imaged similarly to FRAP experiments. User-defined regions at contacts and Z-discs were photoconverted by 300 ms exposure to a 405 laser and the dynamics of the photoconverted protein tracked every 10 s over 5 min. Photoconversion data was quantified in ImageJ (NIH) and changes in signal intensity measured over time in Excel (Microsoft) and plotted in Prism (GraphPad). To establish the photoconverted signal range for each event, the signal intensity of the red photoconverted protein in the region of interest was measured just before and immediately after exposure to the 405 laser. Photoconverted signal was also measured at a Z-disc and similar sized cytoplasmic region 2–3 μm from the photoconverted region over the course of the experiment. Changes in signal intensity of these proximal regions relative to the photoconverted region were plotted over time.

### Electron microscopy

Cardiomyocytes were cultured on collagen-coated MatTek dishes and fixed as described above. After fixation and washing, cells were incubated with 1% OsO_4_ for 1 h. After several PBS washes, dishes were dehydrated through a graded series of 30% to 100% ethanol, and then infiltrated for 1 h in Polybed 812 epoxy resin (Polysciences). After several changes of 100% resin over 24 h, cells were embedded in inverted Beem capsules, cured at 37°C overnight and then hardened for 2 days at 65°C. Blocks were removed from the glass dish via a freeze-thaw method by alternating liquid nitrogen and 100°C water. Ultrathin (60 nm) sections were collected onto 200-mesh copper grids, stained with 2% uranyl acetate in 50% methanol for 10 min and 1% lead citrate for 7 min. Samples were photographed with a JEOL JEM 1400 PLUS transmission electron microscope at 80 kV with a Hamamatsu ORCA-HR side mount camera.

For platinum replica electron microscopy (PREM), cardiomyocytes were first washed with PBS and then extracted for 3 min in PHEM buffer (without fixative) plus 1% Triton X-100 and 10 µM phalloidin (unlabeled). Following extraction, cells were washed 3× in PHEM buffer (without fixative) plus 5 µM phalloidin and fixed for 20 min in PHEM buffer plus 2% glutaraldehyde. Cells were washed and stored in PBS at 4°C until processing. Fixed samples were processed for PREM as described (Svitkina and Borisy, 1998). Replicas were imaged in grids on the JEOL JEM 1400 PLUS transmission electron microscope described above.

### Adenovirus production

Mouse *Cdh2* ORF was first amplified from CDH2-EGFP using PCR and cloned into MCS-BioID2-HA to fuse BioID2 to the C-terminal tail of N-cadherin. The CDH2–BioID2 fusion was then subcloned into pAdTrack-CMV plasmid. Recombinant adenovirus was produced by transforming the pAdTrack-CMV-CDH2-BioID2 plasmid into pAdEasier-1 *E. coli* cells (deposited by Bert Vogelstein, Addgene 16399) (He et al., 1998). Virus packaging and amplification were performed according to the protocol described by Luo et al. (2007). Virus particles were purified using Vivapure AdenoPACK 20 Adenovirus (Ad5) purification and concentration kit (Sartorius). Adeno-X qPCR Titration Kit (Clontech) was used to calculate virus titer using quantitative PCR on an Applied Biosystems 7900HT.

### Adenovirus infection and biotin labeling

Each experimental replicate included four 35 mm dishes with 1×10^6^ cells each (4×10^6^ total). Cardiomyocytes were infected one day after plating with CDH2–BioID2 adenovirus at a MOI of 2. Twenty-four hours later (48 h post-plating), the media was replaced with fresh maintenance media plus 50 µM biotin in both CDH2–BioID2-infected and control uninfected samples. The next day (72 h post-plating), cells were harvested for protein isolation and mass spectrometry. Cell lysate preparation and affinity purification were performed according to published protocols (Kim et al., 2016a; Le Sage et al., 2016).

### Western blotting

Protein samples were separated on an 8% SDS-PAGE gel and transferred onto a PVDF membrane (Bio-Rad). The membrane was blocked in TBST+5% BSA, washed in TBST, incubated with IRDye 680RD Streptavidin (1:1000, LI-COR) in TBST, washed twice in TBST and washed once in PBS. The membrane was scanned using a LI-COR Odyssey Infrared Imager.

### Mass spectrometry and statistical analysis

All protein samples were run on precast Mini-PROTEAN TGX 10% SDS-PAGE gels (Bio-Rad) at 120 V for 5 min so that the proteins migrated into the gel about 1 cm^2^. Gels were stained in Coomassie Blue and a single, ∼1 cm gel slice was excised for each sample and submitted for processing. Excised bands were digested with trypsin as previously described (Braganza et al., 2017). Briefly, the excised gel bands were destained with 25 mM ammonium bicarbonate in 50% acetonitrile (ACN) until no visual stain remained and the gel pieces were dehydrated with 100% ACN. Disulfide bonds were reduced in 10 mM dithiothreitol (DTT, Sigma-Aldrich) at 56°C for 1 h and alkylated with 55 mM iodoacetamide (IAA, Sigma-Aldrich) for 45 min at room temperature in the dark. Excess DTT and IAA were removed by dehydration in 100% ACN before rehydration in 20 ng/µl trypsin (sequencing grade modified, Promega) in 25 mM ammonium bicarbonate and digested overnight at 37°C. The peptides were extracted from gel pieces in a solution containing 70% ACN, 5% formic acid and desalted with Pierce C18 Spin Columns (Thermo Fisher Scientific) according to manufacturer's protocol, dried in a vacuum centrifuge and resuspended in 18 µl of 0.1% formic acid. A pooled instrument control (PIC) sample was prepared by combining 4 µl from each of the 12 samples and used to monitor instrument reproducibility.

Tryptic peptides were analyzed using nano-scale liquid chromatographic tandem mass spectrometry (nLC-MS/MS) using a nanoACQUITY (Waters Corporation) online coupled with an Orbitrap Velos Pro hybrid ion trap mass spectrometer (Thermo Fisher Scientific). For nLC-MS/MS analysis, 1 μl aliquots of each tryptic digest were loaded onto an integrated PicoChip (New Objective) configured with a 25 cm × 75 μm capillary column packed with 3 μm × 120 Å Reprosil C18 media (Waters) and 15 μm pulled tip electrospray emitter. Peptides were eluted off to the mass spectrometer with a 66 min linear gradient of 2–35% ACN plus 0.1% formic acid at a flow rate of 300 nl/min. The full scan MS spectra were collected over mass range m/z 375–1800 in positive ion mode with an FTMS resolution setting of 60,000 at m/z 400 and AGC target 1,000,000 ms. The top 13 most intense ions were sequentially isolated for collision-induced dissociation (CID) tandem mass spectrometry (MS/MS) in the ion trap with ITMS AGC target 5000 ms. Dynamic exclusion (90 s) was enabled to minimize the redundant selection of peptides previously selected for MS/MS fragmentation.

The nLC-MS/MS data were analyzed with MaxQuant software (Cox and Mann, 2008; Tyanova et al., 2016), version 1.6.0.1. Briefly, the proteomic features were quantified using high-resolution full MS intensities after retention alignment and the corresponding MS/MS spectra were searched with Andromeda search engine against the Uniprot mouse database (release November 2017, 82,555 entries) (The UniProt Consortium, 2011). The mass tolerance was set at 20 ppm for the precursor ions and 0.8 Da for the ITMS fragment ions. Andromeda search included specific trypsin enzyme with maximum two missed cleavages, minimum of seven amino acids in length. Fixed modification carbamidomethyl (C), and variable modifications of oxidation (M), acetyl (Protein N-term), and deamidation (NQ) were considered. Protein identification threshold was set to 1% false discovery rate (FDR) as described previously (Cox and Mann, 2008).

Proteins that exhibit statistically significant abundance between CDH2–BioID2 to control were selected as follows. Proteins with a single peptide identification were excluded from the data analysis and Student's *t*-test on log_2_-transformed protein intensity was used for the statistical inference to select CDH2–BioID2-associated proteins. A protein was considered a significant candidate if the *t*-test *P*-value, the probability that an observed match was random, was <0.001 and the fold­-change was >10 when compared to the control.

As a surrogate for protein abundance, MaxQuant iBAQ values were used for label-free absolute quantification of identified proteins (Schwanhäusser et al., 2011). The average iBAQ value for each protein was determined from the six replicates in the both CDH2 and control samples. The final iBAQ value (provided in Table S1) was determined by subtracting the control average from the CDH2 average.

### Bioinformatics analysis

CDH1 BioID proximity proteomics results were from two previous studies (Guo et al., 2014; Van Itallie et al., 2014). The ICD protein list was from a previous curation (Estigoy et al., 2009). Venn diagrams comparing the protein lists were generated using BioVenn (Hulsen et al., 2008). Pathway and disease and function enrichment analysis was performed using Ingenuity Pathway Analysis (IPA) tools (Qiagen). Gene expression data for the identification of HEGs (heart enriched genes) or CEGs (cardiomyocyte enriched genes) were from previous studies (Fagerberg et al., 2014; Li et al., 2017; Tompkins et al., 2016). The heart or cardiomyocyte enriched genes were identified using the Gene Expression Pattern Analyzer (GEPA) algorithm at the threshold of fold-change ≥2 (≥2.5 for cardiovascular differentiation from human embryonic cell data set) (Li et al., 2015). Three types of expression patterns were selected: (1) exclusive high expression in cardiomyocytes or heart; (2) multiple high expression tissues/cells including heart/cardiomyocytes in which the sum of fragment per kilobase of exon per million reads (FPKM) was greater than the total sample number and the number of pattern samples was no greater than 4; and (3) ‘gradient’ pattern with the highest expression in heart/cardiomyocytes and fold-change of the highest and lowest expression is no less than 4. Fisher's exact tests for overrepresentation analysis of HEGs or CEGs were performed using R (https://www.r-project.org/).

### Protein network analysis

The protein interaction map was generated using Ingenuity Pathway Analysis (IPA, Qiagen). Only protein–protein interactions supported by published, experimental data in the manually curated Ingenuity Knowledge Base were considered to build the network. Hierarchical classification was done by grouping the proteins manually using CDH2 at the core. Proteins that bind CDH2 directly were designated as primary interactors. Proteins that bind to primary interactors but not CDH2 were classified as secondary interactors. Proteins that bind secondary interactors were designated as tertiary interactors. Finally, proteins that bind tertiary interactors or to outermost tier proteins were defined as quaternary interactors. 52 proteins could not be linked to the protein network.

## Supplementary Material

Supplementary information

## References

[JCS221606C1] Angst B. D., Khan L. U. R., Severs N. J., Whitely K., Rothery S., Thompson R. P., Magee A. I. and Gourdie R. G. (1997). Dissociated spatial patterning of gap junctions and cell adhesion junctions during postnatal differentiation of ventricular myocardium. *Circ. Res.* 80, 88-94. 10.1161/01.RES.80.1.88 8978327

[JCS221606C2] Barton L. J., Soshnev A. A. and Geyer P. K. (2015). Networking in the nucleus: a spotlight on LEM-domain proteins. *Curr. Opin. Cell Biol.* 34, 1-8. 10.1016/j.ceb.2015.03.005 25863918 PMC4522374

[JCS221606C3] Bass-Zubek A. E., Godsel L. M., Delmar M. and Green K. J. (2009). Plakophilins: multifunctional scaffolds for adhesion and signaling. *Curr. Opin. Cell Biol.* 21, 708-716. 10.1016/j.ceb.2009.07.002 19674883 PMC3091506

[JCS221606C4] Bennett P. M., Maggs A. M., Baines A. J. and Pinder J. C. (2006). The transitional junction: a new functional subcellular domain at the intercalated disc. *Mol. Biol. Cell* 17, 2091-2100. 10.1091/mbc.e05-12-1109 16481394 PMC1415289

[JCS221606C5] Borrmann C. M., Grund C., Kuhn C., Hofmann I., Pieperhoff S. and Franke W. W. (2006). The area composita of adhering junctions connecting heart muscle cells of vertebrates. II. Colocalizations of desmosomal and fascia adhaerens molecules in the intercalated disk. *Eur. J. Cell Biol.* 85, 469-485. 10.1016/j.ejcb.2006.02.009 16600422

[JCS221606C6] Braganza A., Li J., Zeng X., Yates N. A., Dey N. B., Andrews J., Clark J., Zamani L., Wang X.-H., St Croix C. et al. (2017). UBE3B Is a calmodulin-regulated, mitochondrion-associated E3 ubiquitin ligase. *J. Biol. Chem.* 292, 2470-2484. 10.1074/jbc.M116.766824 28003368 PMC5313114

[JCS221606C7] Cartegni L., di Barletta M. R., Barresi R., Squarzoni S., Sabatelli P., Maraldi N., Mora M., Di Blasi C., Cornelio F., Merlini L. et al. (1997). Heart-specific localization of emerin: new insights into Emery-Dreifuss muscular dystrophy. *Hum. Mol. Genet.* 6, 2257-2264. 10.1093/hmg/6.13.2257 9361031

[JCS221606C8] Charras G. and Yap A. S. (2018). Tensile forces and mechanotransduction at cell-cell junctions. *Curr. Biol.* 28, R445-R457. 10.1016/j.cub.2018.02.003 29689229

[JCS221606C9] Chen C. L. and Perrimon N. (2017). Proximity-dependent labeling methods for proteomic profiling in living cells. *Wiley Interdiscip. Rev. Dev. Biol.* 6, e272 10.1002/wdev.272 PMC5553119 28387482

[JCS221606C10] Choi H.-J., Gross J. C., Pokutta S. and Weis W. I. (2009). Interactions of plakoglobin and beta-catenin with desmosomal cadherins: basis of selective exclusion of alpha- and beta-catenin from desmosomes. *J. Biol. Chem.* 284, 31776-31788. 10.1074/jbc.M109.047928 19759396 PMC2797248

[JCS221606C11] Cox J. and Mann M. (2008). MaxQuant enables high peptide identification rates, individualized p.p.b.-range mass accuracies and proteome-wide protein quantification. *Nat. Biotechnol.* 26, 1367-1372. 10.1038/nbt.1511 19029910

[JCS221606C12] Drees F., Pokutta S., Yamada S., Nelson W. J. and Weis W. I. (2005). Alpha-catenin is a molecular switch that binds E-cadherin-beta-catenin and regulates actin-filament assembly. *Cell* 123, 903-915. 10.1016/j.cell.2005.09.021 16325583 PMC3369825

[JCS221606C13] Ehler E. (2016). Cardiac cytoarchitecture - why the “hardware” is important for heart function! *Biochim. Biophys. Acta* 1863, 1857-1863. 10.1016/j.bbamcr.2015.11.006 26577135 PMC5104690

[JCS221606C14] Ehler E. (2018). Actin-associated proteins and cardiomyopathy-the ‘unknown’ beyond troponin and tropomyosin. *Biophys. Rev.* 10 1121-1128. 10.1007/s12551-018-0428-1 29869751 PMC6082317

[JCS221606C15] Ehler E., Moore-Morris T. and Lange S. (2013). Isolation and culture of neonatal mouse cardiomyocytes. *J. Vis. Exp.* 79, e50154 10.3791/50154 PMC3857885 24056408

[JCS221606C16] Estigoy C. B., Pontén F., Odeberg J., Herbert B., Guilhaus M., Charleston M., Ho J. W. K., Cameron D. and Dos Remedios C. G. (2009). Intercalated discs: multiple proteins perform multiple functions in non-failing and failing human hearts. *Biophys. Rev.* 1, 43 10.1007/s12551-008-0007-y 28510153 PMC5418371

[JCS221606C17] Fagerberg L., Hallström B. M., Oksvold P., Kampf C., Djureinovic D., Odeberg J., Habuka M., Tahmasebpoor S., Danielsson A., Edlund K. et al. (2014). Analysis of the human tissue-specific expression by genome-wide integration of transcriptomics and antibody-based proteomics. *Mol. Cell. Proteomics* 13, 397-406. 10.1074/mcp.M113.035600 24309898 PMC3916642

[JCS221606C18] Fan W., Tang Z., Chen D., Moughon D., Ding X., Chen S., Zhu M. and Zhong Q. (2010). Keap1 facilitates p62-mediated ubiquitin aggregate clearance via autophagy. *Autophagy* 6, 614-621. 10.4161/auto.6.5.12189 20495340 PMC4423623

[JCS221606C19] Faulkner G., Pallavicini A., Formentin E., Comelli A., Ievolella C., Trevisan S., Bortoletto G., Scannapieco P., Salamon M., Mouly V. et al. (1999). ZASP: a new Z-band alternatively spliced PDZ-motif protein. *J. Cell Biol.* 146, 465-475. 10.1083/jcb.146.2.465 10427098 PMC3206570

[JCS221606C20] Frank D. and Frey N. (2011). Cardiac Z-disc signaling network. *J. Biol. Chem.* 286, 9897-9904. 10.1074/jbc.R110.174268 21257757 PMC3060542

[JCS221606C21] Frank D., Kuhn C., Katus H. A. and Frey N. (2006). The sarcomeric Z-disc: a nodal point in signalling and disease. *J. Mol. Med. (Berl.)* 84, 446-468. 10.1007/s00109-005-0033-1 16416311

[JCS221606C22] Franke W. W., Borrmann C. M., Grund C. and Pieperhoff S. (2006). The area composita of adhering junctions connecting heart muscle cells of vertebrates. I. Molecular definition in intercalated disks of cardiomyocytes by immunoelectron microscopy of desmosomal proteins. *Eur. J. Cell Biol.* 85, 69-82. 10.1016/j.ejcb.2005.11.003 16406610

[JCS221606C23] Franke W. W., Schumacher H., Borrmann C. M., Grund C., Winter-Simanowski S., Schlechter T., Pieperhoff S. and Hofmann I. (2007). The area composita of adhering junctions connecting heart muscle cells of vertebrates - III: assembly and disintegration of intercalated disks in rat cardiomyocytes growing in culture. *Eur. J. Cell Biol.* 86, 127-142. 10.1016/j.ejcb.2006.11.003 17275137

[JCS221606C24] Garcia M. A., Nelson W. J. and Chavez N. (2018). Cell-cell junctions organize structural and signaling networks. *Cold Spring Harb. Perspect. Biol.* 10, a029181 10.1101/cshperspect.a029181 28600395 PMC5773398

[JCS221606C25] Garcia-Gras E., Lombardi R., Giocondo M. J., Willerson J. T., Schneider M. D., Khoury D. S. and Marian A. J. (2006). Suppression of canonical Wnt/beta-catenin signaling by nuclear plakoglobin recapitulates phenotype of arrhythmogenic right ventricular cardiomyopathy. *J. Clin. Invest.* 116, 2012-2021. 10.1172/JCI27751 16823493 PMC1483165

[JCS221606C26] Geraldo S., Khanzada U. K., Parsons M., Chilton J. K. and Gordon-Weeks P. R. (2008). Targeting of the F-actin-binding protein drebrin by the microtubule plus-tip protein EB3 is required for neuritogenesis. *Nat. Cell Biol.* 10, 1181-1189. 10.1038/ncb1778 18806788

[JCS221606C27] Goncharova E. J., Kam Z. and Geiger B. (1992). The involvement of adherens junction components in myofibrillogenesis in cultured cardiac myocytes. *Development* 114, 173-183. 1576958 10.1242/dev.114.1.173

[JCS221606C28] Goossens S., Janssens B., Bonne S., De Rycke R., Braet F., van Hengel J. and van Roy F. (2007). A unique and specific interaction between alphaT-catenin and plakophilin-2 in the area composita, the mixed-type junctional structure of cardiac intercalated discs. *J. Cell Sci.* 120, 2126-2136. 10.1242/jcs.004713 17535849

[JCS221606C29] Guo Z., Neilson L. J., Zhong H., Murray P. S., Zanivan S. and Zaidel-Bar R. (2014). E-cadherin interactome complexity and robustness resolved by quantitative proteomics. *Sci. Signal.* 7, rs7 10.1126/scisignal.2005473 25468996 PMC4972397

[JCS221606C30] Halbleib J. M. and Nelson W. J. (2006). Cadherins in development: cell adhesion, sorting, and tissue morphogenesis. *Genes Dev.* 20, 3199-3214. 10.1101/gad.1486806 17158740

[JCS221606C31] Hazan R. B., Kang L., Roe S., Borgen P. I. and Rimm D. L. (1997). Vinculin is associated with the E-cadherin adhesion complex. *J. Biol. Chem.* 272, 32448-32453. 10.1074/jbc.272.51.32448 9405455

[JCS221606C32] He T.-C., Zhou S., da Costa L. T., Yu J., Kinzler K. W. and Vogelstein B. (1998). A simplified system for generating recombinant adenoviruses. *Proc. Natl. Acad. Sci. USA* 95, 2509-2514. 10.1073/pnas.95.5.2509 9482916 PMC19394

[JCS221606C33] Herzog W. (2018). The multiple roles of titin in muscle contraction and force production. *Biophys. Rev.* 10, 1187-1199. 10.1007/s12551-017-0395-y 29353351 PMC6082311

[JCS221606C34] Hirschy A., Schatzmann F., Ehler E. and Perriard J.-C. (2006). Establishment of cardiac cytoarchitecture in the developing mouse heart. *Dev. Biol.* 289, 430-441. 10.1016/j.ydbio.2005.10.046 16337936

[JCS221606C35] Hishiya A., Kitazawa T. and Takayama S. (2010). BAG3 and Hsc70 interact with actin capping protein CapZ to maintain myofibrillar integrity under mechanical stress. *Circ. Res.* 107, 1220-1231. 10.1161/CIRCRESAHA.110.225649 20884878 PMC2980587

[JCS221606C36] Hoffman B. D. and Yap A. S. (2015). Towards a dynamic understanding of cadherin-based mechanobiology. *Trends Cell Biol.* 25, 803-814. 10.1016/j.tcb.2015.09.008 26519989

[JCS221606C37] Hulsen T., de Vlieg J. and Alkema W. (2008). BioVenn - a web application for the comparison and visualization of biological lists using area-proportional Venn diagrams. *BMC Genomics* 9, 488 10.1186/1471-2164-9-488 18925949 PMC2584113

[JCS221606C38] Katsamba P., Carroll K., Ahlsen G., Bahna F., Vendome J., Posy S., Rajebhosale M., Price S., Jessell T. M., Ben-Shaul A. et al. (2009). Linking molecular affinity and cellular specificity in cadherin-mediated adhesion. *Proc. Natl. Acad. Sci. USA* 106, 11594-11599. 10.1073/pnas.0905349106 19553217 PMC2710653

[JCS221606C39] Kim D. I., Jensen S. C., Noble K. A., Kc B., Roux K. H., Motamedchaboki K. and Roux K. J. (2016a). An improved smaller biotin ligase for BioID proximity labeling. *Mol. Biol. Cell* 27, 1188-1196. 10.1091/mbc.E15-12-0844 26912792 PMC4831873

[JCS221606C40] Kim E., Ilic N., Shrestha Y., Zou L., Kamburov A., Zhu C., Yang X., Lubonja R., Tran N., Nguyen C. et al. (2016b). Systematic functional interrogation of rare cancer variants identifies oncogenic alleles. *Cancer Discov.* 6, 714-726. 10.1158/2159-8290.CD-16-0160 27147599 PMC4930723

[JCS221606C41] Kostetskii I., Li J., Xiong Y., Zhou R., Ferrari V. A., Patel V. V., Molkentin J. D. and Radice G. L. (2005). Induced deletion of the N-cadherin gene in the heart leads to dissolution of the intercalated disc structure. *Circ. Res.* 96, 346-354. 10.1161/01.RES.0000156274.72390.2c 15662031

[JCS221606C42] Le Sage V., Cinti A. and Mouland A. J. (2016). Proximity-dependent biotinylation for identification of interacting proteins. *Curr. Protoc. Cell Biol.* 73, 17.19.1-17.19.12. 10.1002/cpcb.11 27906451

[JCS221606C43] Li J., Patel V. V., Kostetskii I., Xiong Y., Chu A. F., Jacobson J. T., Yu C., Morley G. E., Molkentin J. D. and Radice G. L. (2005). Cardiac-specific loss of N-cadherin leads to alteration in connexins with conduction slowing and arrhythmogenesis. *Circ. Res.* 97, 474-481. 10.1161/01.RES.0000181132.11393.18 16100040

[JCS221606C44] Li D., Zhang W., Liu Y., Haneline L. S. and Shou W. (2012a). Lack of plakoglobin in epidermis leads to keratoderma. *J. Biol. Chem.* 287, 10435-10443. 10.1074/jbc.M111.299669 22315228 PMC3322998

[JCS221606C45] Li J., Goossens S., van Hengel J., Gao E., Cheng L., Tyberghein K., Shang X., De Rycke R., van Roy F. and Radice G. L. (2012b). Loss of alphaT-catenin alters the hybrid adhering junctions in the heart and leads to dilated cardiomyopathy and ventricular arrhythmia following acute ischemia. *J. Cell Sci.* 125, 1058-1067. 10.1242/jcs.098640 22421363 PMC3311935

[JCS221606C46] Li Y., Lin B. and Yang L. (2015). Comparative transcriptomic analysis of multiple cardiovascular fates from embryonic stem cells predicts novel regulators in human cardiogenesis. *Sci. Rep.* 5, 9758 10.1038/srep09758 25997157 PMC4440522

[JCS221606C47] Li B., Qing T., Zhu J., Wen Z., Yu Y., Fukumura R., Zheng Y., Gondo Y. and Shi L. (2017). A comprehensive mouse transcriptomic BodyMap across 17 tissues by RNA-seq. *Sci. Rep.* 7, 4200 10.1038/s41598-017-04520-z 28646208 PMC5482823

[JCS221606C48] Luo Y. and Radice G. L. (2003). Cadherin-mediated adhesion is essential for myofibril continuity across the plasma membrane but not for assembly of the contractile apparatus. *J. Cell Sci.* 116, 1471-1479. 10.1242/jcs.00339 12640032

[JCS221606C49] Luo Y., Ferreira-Cornwell M., Baldwin H., Kostetskii I., Lenox J., Lieberman M. and Radice G. (2001). Rescuing the N-cadherin knockout by cardiac-specific expression of N- or E-cadherin. *Development* 128, 459-469. 11171330 10.1242/dev.128.4.459

[JCS221606C50] Luo J., Deng Z.-L., Luo X., Tang N., Song W.-X., Chen J., Sharff K. A., Luu H. H., Haydon R. C., Kinzler K. W. et al. (2007). A protocol for rapid generation of recombinant adenoviruses using the AdEasy system. *Nat. Protoc.* 2, 1236-1247. 10.1038/nprot.2007.135 17546019

[JCS221606C51] Moncman C. L. and Wang K. (1995). Nebulette: a 107 kD nebulin-like protein in cardiac muscle. *Cell Motil. Cytoskeleton* 32, 205-225. 10.1002/cm.970320305 8581976

[JCS221606C52] Mueller F., Morisaki T., Mazza D. and McNally J. G. (2012). Minimizing the impact of photoswitching of fluorescent proteins on FRAP analysis. *Biophys. J.* 102, 1656-1665. 10.1016/j.bpj.2012.02.029 22500766 PMC3318116

[JCS221606C53] Nakata S., Fujita N., Kitagawa Y., Okamoto R., Ogita H. and Takai Y. (2007). Regulation of platelet-derived growth factor receptor activation by afadin through SHP-2: implications for cellular morphology. *J. Biol. Chem.* 282, 37815-37825. 10.1074/jbc.M707461200 17971444

[JCS221606C54] Nekrasova O. and Green K. J. (2013). Desmosome assembly and dynamics. *Trends Cell Biol.* 23, 537-546. 10.1016/j.tcb.2013.06.004 23891292 PMC3913269

[JCS221606C55] Oh S. W., Pope R. K., Smith K. P., Crowley J. L., Nebl T., Lawrence J. B. and Luna E. J. (2003). Archvillin, a muscle-specific isoform of supervillin, is an early expressed component of the costameric membrane skeleton. *J. Cell Sci.* 116, 2261-2275. 10.1242/jcs.00422 12711699

[JCS221606C56] Padmanabhan A., Rao M. V., Wu Y. and Zaidel-Bar R. (2015). Jack of all trades: functional modularity in the adherens junction. *Curr. Opin. Cell Biol.* 36, 32-40. 10.1016/j.ceb.2015.06.008 26189061

[JCS221606C57] Perrin B. J., Amann K. J. and Huttenlocher A. (2006). Proteolysis of cortactin by calpain regulates membrane protrusion during cell migration. *Mol. Biol. Cell* 17, 239-250. 10.1091/mbc.e05-06-0488 16280362 PMC1345662

[JCS221606C58] Pieperhoff S. and Franke W. W. (2007). The area composita of adhering junctions connecting heart muscle cells of vertebrates - IV: coalescence and amalgamation of desmosomal and adhaerens junction components - late processes in mammalian heart development. *Eur. J. Cell Biol.* 86, 377-391. 10.1016/j.ejcb.2007.04.001 17532539

[JCS221606C59] Pokutta S., Drees F., Takai Y., Nelson W. J. and Weis W. I. (2002). Biochemical and structural definition of the l-afadin- and actin-binding sites of alpha-catenin. *J. Biol. Chem.* 277, 18868-18874. 10.1074/jbc.M201463200 11907041 PMC3368618

[JCS221606C60] Pokutta S., Choi H.-J., Ahlsen G., Hansen S. D. and Weis W. I. (2014). Structural and thermodynamic characterization of cadherin beta-catenin alpha-catenin complex formation. *J. Biol. Chem.* 289, 13589-13601. 10.1074/jbc.M114.554709 24692547 PMC4036364

[JCS221606C61] Ratheesh A. and Yap A. S. (2012). A bigger picture: classical cadherins and the dynamic actin cytoskeleton. *Nat. Rev. Mol. Cell Biol.* 13, 673-679. 10.1038/nrm3431 22931853

[JCS221606C62] Rimm D. L., Koslov E. R., Kebriaei P., Cianci C. D. and Morrow J. S. (1995). Alpha 1(E)-catenin is an actin-binding and -bundling protein mediating the attachment of F-actin to the membrane adhesion complex. *Proc. Natl. Acad. Sci. USA* 92, 8813-8817. 10.1073/pnas.92.19.8813 7568023 PMC41057

[JCS221606C63] Sawyer J. K., Harris N. J., Slep K. C., Gaul U. and Peifer M. (2009). The Drosophila afadin homologue Canoe regulates linkage of the actin cytoskeleton to adherens junctions during apical constriction. *J. Cell Biol.* 186, 57-73. 10.1083/jcb.200904001 19596848 PMC2712996

[JCS221606C64] Schwanhäusser B., Busse D., Li N., Dittmar G., Schuchhardt J., Wolf J., Chen W. and Selbach M. (2011). Global quantification of mammalian gene expression control. *Nature* 473, 337-342. 10.1038/nature10098 21593866

[JCS221606C65] Shafraz O., Rubsam M., Stahley S. N., Caldara A. L., Kowalczyk A. P., Niessen C. M. and Sivasankar S. (2018). E-cadherin binds to desmoglein to facilitate desmosome assembly. *eLife* 7, e37629 10.7554/eLife.37629 29999492 PMC6066328

[JCS221606C66] Sheikh F., Chen Y., Liang X., Hirschy A., Stenbit A. E., Gu Y., Dalton N. D., Yajima T., Lu Y., Knowlton K. U. et al. (2006). alpha-E-catenin inactivation disrupts the cardiomyocyte adherens junction, resulting in cardiomyopathy and susceptibility to wall rupture. *Circulation* 114, 1046-1055. 10.1161/CIRCULATIONAHA.106.634469 16923756

[JCS221606C67] Svitkina T. M. (2017). Platinum replica electron microscopy: imaging the cytoskeleton globally and locally. *Int. J. Biochem. Cell Biol.* 86, 37-41. 10.1016/j.biocel.2017.03.009 28323208 PMC5424547

[JCS221606C68] Svitkina T. M. and Borisy G. G. (1998). Correlative light and electron microscopy of the cytoskeleton of cultured cells. *Methods Enzymol.* 298, 570-592. 10.1016/S0076-6879(98)98045-4 9751908

[JCS221606C69] Tachibana K., Nakanishi H., Mandai K., Ozaki K., Ikeda W., Yamamoto Y., Nagafuchi A., Tsukita S. and Takai Y. (2000). Two cell adhesion molecules, nectin and cadherin, interact through their cytoplasmic domain-associated proteins. *J. Cell Biol.* 150, 1161-1176. 10.1083/jcb.150.5.1161 10974003 PMC2175253

[JCS221606C70] The UniProt Consortium (2011). Ongoing and future developments at the Universal Protein Resource. *Nucleic Acids Res.* 39, D214-D219. 10.1093/nar/gkq1020 21051339 PMC3013648

[JCS221606C71] Tompkins J. D., Jung M., Chen C.-Y., Lin Z., Ye J., Godatha S., Lizhar E., Wu X., Hsu D., Couture L. A. et al. (2016). Mapping human pluripotent-to-cardiomyocyte differentiation: methylomes, transcriptomes, and exon DNA methylation “Memories”. *EBioMedicine* 4, 74-85. 10.1016/j.ebiom.2016.01.021 26981572 PMC4776252

[JCS221606C72] Tyanova S., Temu T. and Cox J. (2016). The MaxQuant computational platform for mass spectrometry-based shotgun proteomics. *Nat. Protoc.* 11, 2301-2319. 10.1038/nprot.2016.136 27809316

[JCS221606C73] Ueda S., Blee A. M., Macway K. G., Renner D. J. and Yamada S. (2015). Force dependent biotinylation of myosin IIA by alpha-catenin tagged with a promiscuous biotin ligase. *PLoS ONE* 10, e0122886 10.1371/journal.pone.0122886 25806963 PMC4373798

[JCS221606C74] van Hengel J., Calore M., Bauce B., Dazzo E., Mazzotti E., De Bortoli M., Lorenzon A., Li Mura I. E. A., Beffagna G., Rigato I. et al. (2013). Mutations in the area composita protein alphaT-catenin are associated with arrhythmogenic right ventricular cardiomyopathy. *Eur. Heart J.* 34, 201-210. 10.1093/eurheartj/ehs373 23136403

[JCS221606C75] Van Itallie C. M., Tietgens A. J., Aponte A., Fredriksson K., Fanning A. S., Gucek M. and Anderson J. M. (2014). Biotin ligase tagging identifies proteins proximal to E-cadherin, including lipoma preferred partner, a regulator of epithelial cell-cell and cell-substrate adhesion. *J. Cell Sci.* 127, 885-895. 10.1242/jcs.140475 24338363 PMC3924204

[JCS221606C76] Vendome J., Felsovalyi K., Song H., Yang Z., Jin X., Brasch J., Harrison O. J., Ahlsen G., Bahna F., Kaczynska A. et al. (2014). Structural and energetic determinants of adhesive binding specificity in type I cadherins. *Proc. Natl. Acad. Sci. USA* 111, E4175-E4184. 10.1073/pnas.1416737111 25253890 PMC4210030

[JCS221606C77] Vermij S. H., Abriel H. and van Veen T. A. B. (2017). Refining the molecular organization of the cardiac intercalated disc. *Cardiovasc. Res.* 113, 259-275. 10.1093/cvr/cvw259 28069669

[JCS221606C78] Vite A. and Radice G. L. (2014). N-cadherin/catenin complex as a master regulator of intercalated disc function. *Cell Commun. Adhes.* 21, 169-179. 10.3109/15419061.2014.908853 24766605 PMC6054126

[JCS221606C79] Vite A., Li J. and Radice G. L. (2015). New functions for alpha-catenins in health and disease: from cancer to heart regeneration. *Cell Tissue Res.* 360, 773-783. 10.1007/s00441-015-2123-x 25673211 PMC4456210

[JCS221606C80] Wang S., Tukachinsky H., Romano F. B. and Rapoport T. A. (2016). Cooperation of the ER-shaping proteins atlastin, lunapark, and reticulons to generate a tubular membrane network. *eLife* 5, e18605 10.7554/eLife.18605 27619977 PMC5021524

[JCS221606C81] Weins A., Schwarz K., Faul C., Barisoni L., Linke W. A. and Mundel P. (2001). Differentiation- and stress-dependent nuclear cytoplasmic redistribution of myopodin, a novel actin-bundling protein. *J. Cell Biol.* 155, 393-404. 10.1083/jcb.200012039 11673475 PMC2150840

[JCS221606C82] Weiss E. E., Kroemker M., Rüdiger A.-H., Jockusch B. M. and Rüdiger M. (1998). Vinculin is part of the cadherin-catenin junctional complex: complex formation between alpha-catenin and vinculin. *J. Cell Biol.* 141, 755-764. 10.1083/jcb.141.3.755 9566974 PMC2132754

[JCS221606C83] Wickline E. D., Dale I. W., Merkel C. D., Heier J. A., Stolz D. B. and Kwiatkowski A. V. (2016). alphaT-catenin is a constitutive actin-binding alpha-catenin that directly couples the cadherin catenin complex to actin filaments. *J. Biol. Chem.* 291, 15687-15699. 10.1074/jbc.M116.735423 27231342 PMC4957052

[JCS221606C84] Wulfkuhle J. D., Donina I. E., Stark N. H., Pope R. K., Pestonjamasp K. N., Niswonger M. L. and Luna E. J. (1999). Domain analysis of supervillin, an F-actin bundling plasma membrane protein with functional nuclear localization signals. *J. Cell Sci.* 112, 2125-2136. 10362542 10.1242/jcs.112.13.2125

[JCS221606C85] Yamada S., Pokutta S., Drees F., Weis W. I. and Nelson W. J. (2005). Deconstructing the cadherin-catenin-actin complex. *Cell* 123, 889-901. 10.1016/j.cell.2005.09.020 16325582 PMC3368712

[JCS221606C86] Yonemura S., Wada Y., Watanabe T., Nagafuchi A. and Shibata M. (2010). alpha-Catenin as a tension transducer that induces adherens junction development. *Nat. Cell Biol.* 12, 533-542. 10.1038/ncb2055 20453849

[JCS221606C87] Zhang M., Chang H., Zhang Y., Yu J., Wu L., Ji W., Chen J., Liu B., Lu J., Liu Y. et al. (2012). Rational design of true monomeric and bright photoactivatable fluorescent proteins. *Nat. Methods* 9, 727-729. 10.1038/nmeth.2021 22581370

[JCS221606C88] Zheng M., Cheng H., Banerjee I. and Chen J. (2010). ALP/Enigma PDZ-LIM domain proteins in the heart. *J Mol Cell Biol* 2, 96-102. 10.1093/jmcb/mjp038 20042479 PMC2905065

[JCS221606C89] Zuleger N., Kelly D. A., Richardson A. C., Kerr A. R. W., Goldberg M. W., Goryachev A. B. and Schirmer E. C. (2011). System analysis shows distinct mechanisms and common principles of nuclear envelope protein dynamics. *J. Cell Biol.* 193, 109-123. 10.1083/jcb.201009068 21444689 PMC3082195

