# Supplementary material for: The N-cadherin interactome in primary cardiomyocytes as defined using quantitative proximity proteomics
Source: J Cell Sci. 2019 Feb 11;132(3):jcs221606. doi: 10.1242/jcs.221606 (PMC6382013; doi:10.1242/jcs.221606)
Supplement: Supplementary information [file joces-132-221606-s1.pdf]

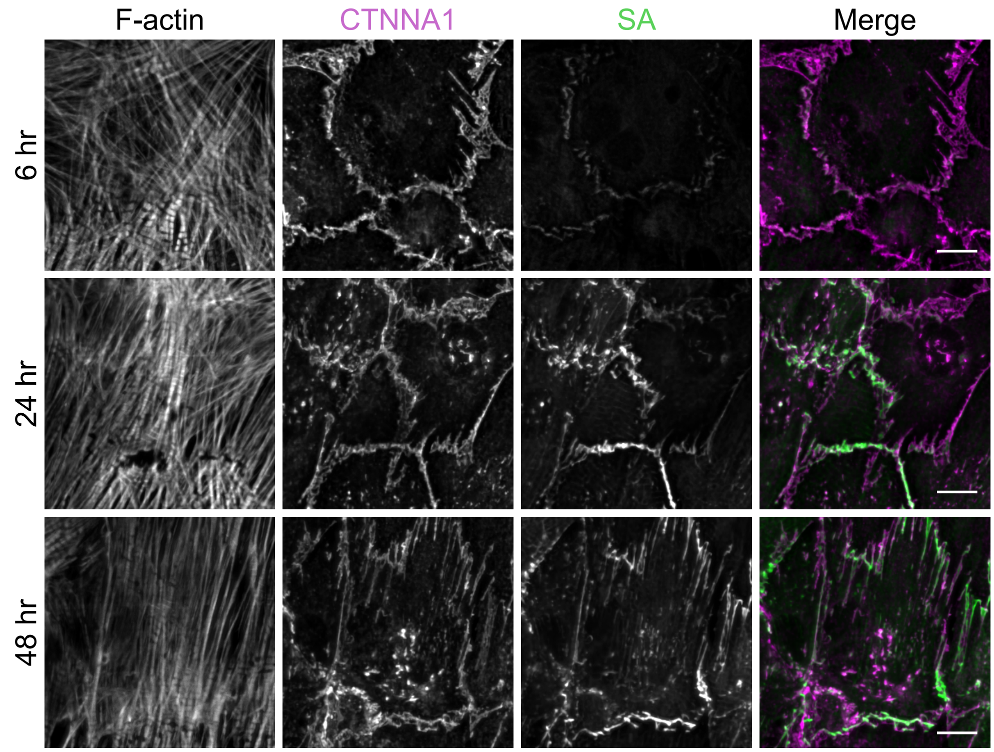

Figure S1. (accompanies Fig 3) Time course of biotin labeling. Cardiomyocytes infected with Cdh2-BioID2 adenovirus were fixed 6, 24 and 48 hours after biotin addition. Cells were stained for F-actin, CTNNA1 and biotin (streptavidin, SA). CTNNA1 (magenta) and SA (green) channels are shown in merge. Scale bar is 10 μm.

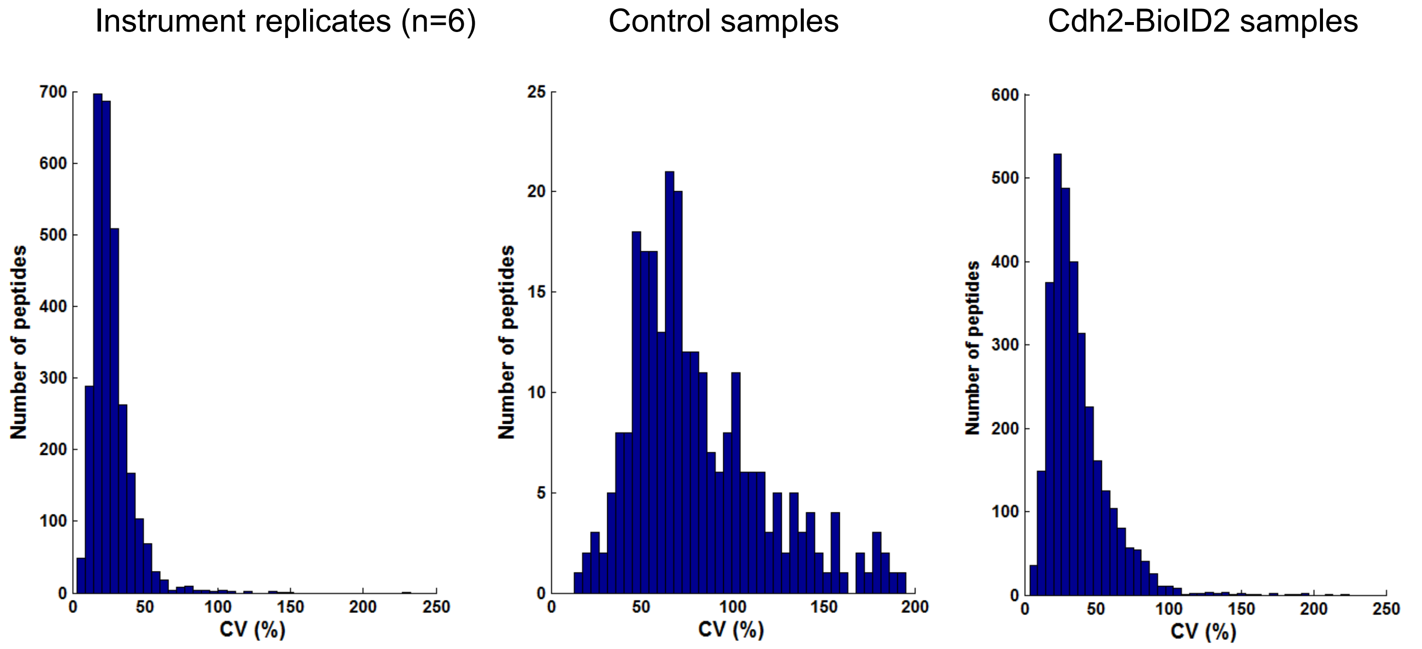

Figure S2. (accompanies Fig 4). A. Coefficient of variance (CV) for mass spec analysis of instrument replicates, control samples and experimental (Cdh2-BioID2) samples. B. Integrin subunit mRNA expression in primary cardiomyocytes determined by quantitative PCR. Graph shows the average mRNA copies per cell of Itgb1, Itgb3, Itgb5, Itga1, Itga5 and Itga7. Errors bars show standard deviation from two replicates. Average RNA copy number is shown above each bar.

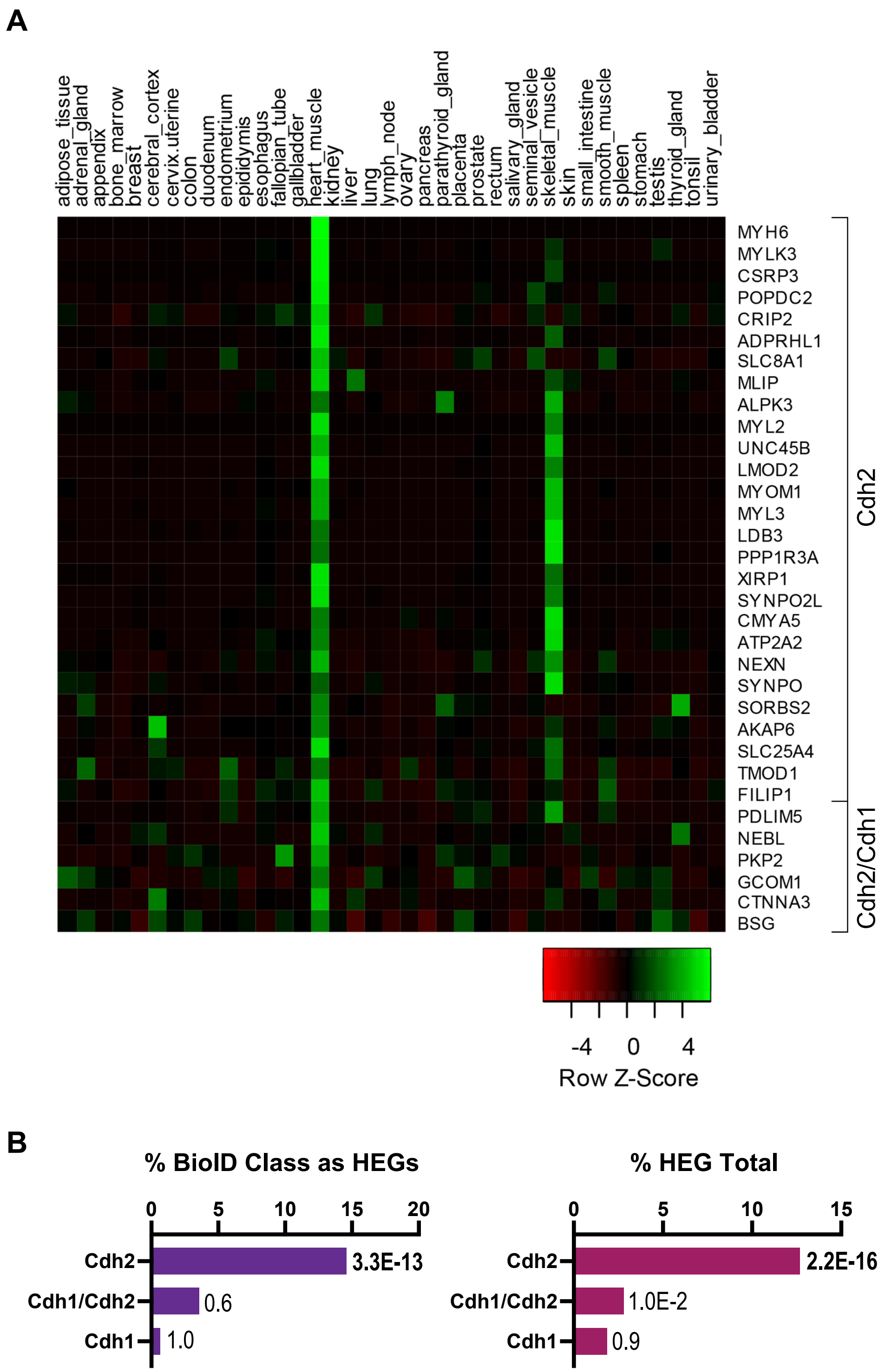

Figure S3. (accompanies Fig. 5) A. Heat map of CDH2 or CDH2/CDH1 expression profiles in human tissues. B. Left, percentage of each BioID class as HEGs. Right, fraction of those BioID HEGs in the total HEG population. P value of Fisher's exact test shown.

Journal of Cell Science • Supplementary information

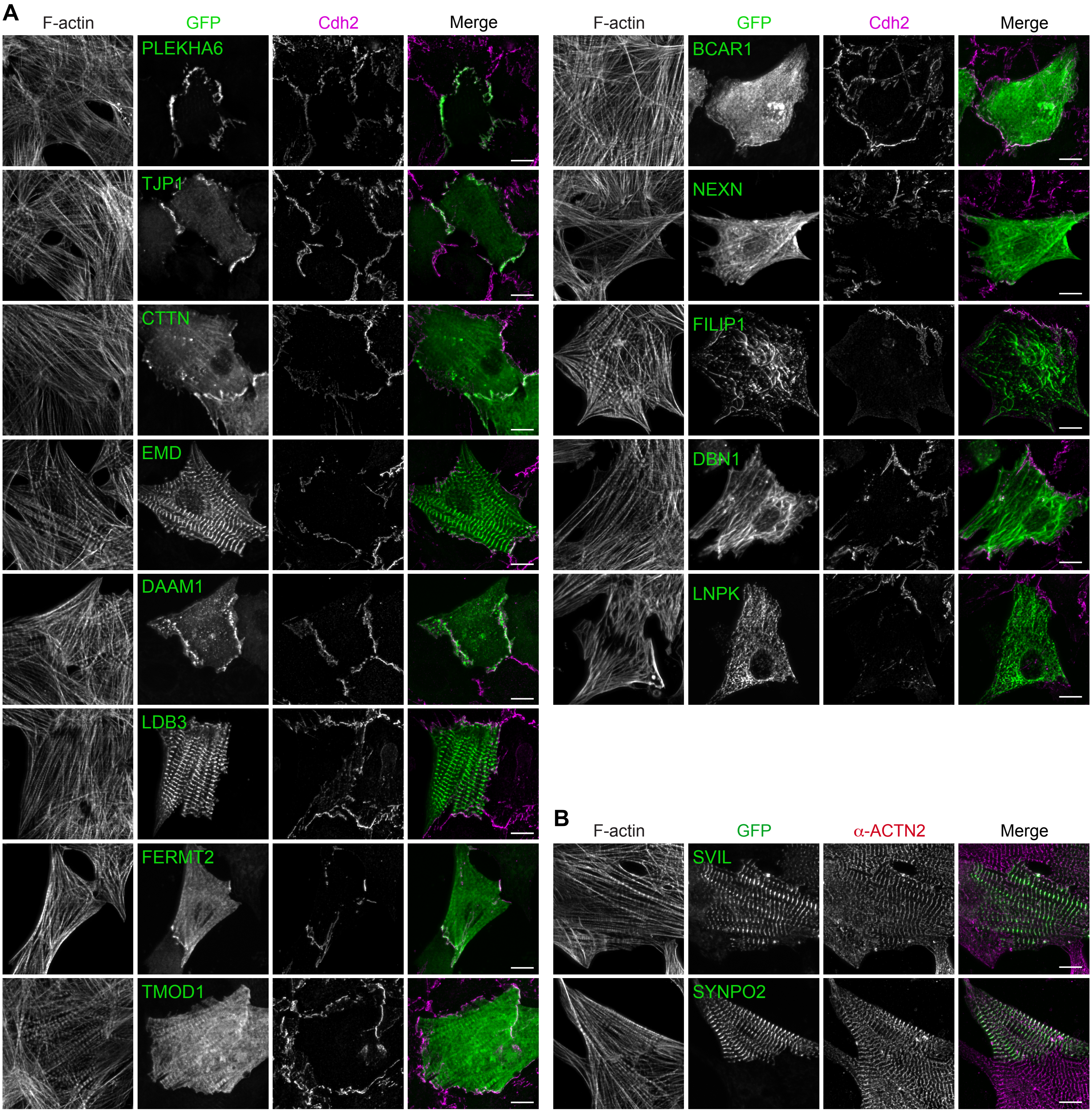

Figure S4. (accompanies Fig. 7). A. CDH2 interactome protein localization. Cardiomyocytes transfected with GFP-tagged Cdh2-BioID hits and stained for Cdh2 and F-actin. Individual and merged GFP (green) and CDH2 (magenta) channels shown. Note that LNPK was tagged with mCherry and DBN1 (paralog of DBNL) was tagged with YFP. Both fusion protein channels were pseudo-colored green for consistent comparison. Tested but not shown are PHLDB1, SQSTM1, TLN1, PARVA, TRIM55, CSRP1, DPYSL3 and COBLL1. All formed aggregates or were cytoplasmic when expressed in cardiomyocytes. B. SVIL and SYNPO2 localize to Z-discs. Cardiomyocytes transfected with EGFP-tagged SVIL and SYNPO2. Cells were fixed 24 hours post-transfection and stained for ACTN2 and F-actin. Scale bar is 10 μm for A and B.

Journal of Cell Science • Supplementary information

Table S1

Click here to Download Table S1

Table S2

Click here to Download Table S2

Table S3

Click here to Download Table S3

Journal of Cell Science • Supplementary information

Table S4. (accompanies Fig. 6) List of unconnected Cdh2-BioID2 hits

PDLIM3
ARHGAP1
ARHGAP23
CAST
EHBP1
EHBP1L1
JCAD
PDAP1
PHACTR2
PLEKHA6
SHB
SLC30A1
SLITRK4
SLK
ADPRHL1
ALPK3
CDV3
CMYA5
CRIP2
ELOB
FILIP1
FILIP1L
FRMD4A
ITM2B
ITM2C
JPH2
LMOD2
LNPK
MAP6
MAST4
MLIP
MYLK3
OBSL1
PCDH7
PGAM2
PPP1R12B
PURB
SHROOM1
SLC25A4
STBD1
SYNPO2L
TBCB
TMCC1
UBAP2L
UNC45B
MURC
GCOM1
PALM2-AKAP2
CCDC9B
C4orf54
GM11639
C10ORF71
